# ADAMTS16 drives epithelial-mesenchymal transition and metastasis through a feedback loop upon TGF-β1 activation in lung adenocarcinoma

**DOI:** 10.1038/s41419-024-07226-z

**Published:** 2024-11-17

**Authors:** Lingyan Xiao, Qian Li, Shuaijun Chen, Yongbiao Huang, Li Ma, Yuan Wang, Junjie Chen, Jun Zhang, Andong Liu, Xianglin Yuan, Yuanhui Liu, Bo Liu

**Affiliations:** 1 Department of Oncology, Tongji Hospital, Tongji Medical College, Huazhong University of Science and Technology, Wuhan, China; 2 Department of Pathophysiology, School of Basic Medicine, Tongji Medical College, Huazhong University of Science and Technology, Wuhan, China; 3 Department of Pathology, The Central Hospital of Wuhan, Tongji Medical College, Huazhong University of Science and Technology, Wuhan, China; 4 Department of Obstetrics and Gynecology, Union Hospital, Tongji Medical College, Huazhong University of Science and Technology, Wuhan, China; 5 Department of Human Anatomy, School of Basic Medicine, Tongji Medical College, Huazhong University of Science and Technology, Wuhan, China

**Keywords:** Cancer, Cell biology

## Abstract

Lung adenocarcinoma (LUAD) is the major subtype of lung cancer. The poor prognosis of LUAD patients is attributed primarily to metastasis. ADAMTS16 is a crucial member of the ADAMTS family and is involved in tumor progression. However, its role and regulatory mechanism in LUAD remain unexplored. In this study, ADAMTS16 was identified as a crucial oncogene and survival predictor in LUAD via analyses of public datasets. Clinical specimens and tissue microarrays confirmed the differential expression and prognostic value of ADAMTS16 in LUAD patients. Transcriptome data and in vitro experiments demonstrated that ADAMTS16 was positively associated with epithelial-mesenchymal transition (EMT) and the migration abilities of LUAD cells. Knockdown of ADAMTS16 attenuated lung and pleural metastasis in an animal model. Mechanistically, the results of the enzyme-linked immunosorbent assay (ELISA) and western blot (WB) suggested that ADAMTS16 activated the TGF-β signaling pathway by facilitating the conversion of LAP-TGF-β1 to active TGF-β1. Co-Immunoprecipitation (co-IP) indicated an interaction between ADAMTS16 and LAP-TGF-β1. Inhibition of ADAMTS16 impaired EMT and aggressiveness of LUAD cells, while treatment with recombinant TGF-β1 reversed this inhibition. Chromatin immunoprecipitation (ChIP) and dual-luciferase reporter assays indicated that SOX4 acted as a transcriptional activator of ADAMTS16 and that TGF-β1 regulated the expression of ADAMTS16 by increasing the binding of SOX4 to the promoter of ADAMTS16. Suppressing the TGF-β signaling pathway inhibited ADAMTS16 expression, EMT, and lung metastasis, whereas overexpressing SOX4 reversed this inhibition. Therefore, ADAMTS16 forms a positive feedback loop with the TGF-β1/SOX4 axis to regulate EMT and metastasis, and disruption of this feedback loop inhibits tumor progression. These findings underscore the potential of ADAMTS16 as a prognostic biomarker and therapeutic target in LUAD and offer novel insight into the mechanism of EMT and metastasis.

## Introduction

Lung cancer is the leading cause of cancer-related mortality worldwide, accounting for 18% of all cancer-related deaths [1]. Lung cancer encompasses various subtypes that have distinct molecular mechanisms and prognostic profiles. LUAD, the most prevalent subtype of non-small cell lung cancer (NSCLC), poses a significant clinical challenge because of its poor prognosis [2]. Despite advances in treatment, only 28% of patients with LUAD survive beyond five years [3]. Cancer-related death is attributed primarily to metastasis, and for patients with NSCLC, the 5-year relative survival rate drastically decreases from 65% for localized tumors to 9% once distant metastasis occurs [3]. The process of metastasis involves EMT wherein cancer cells acquire mesenchymal properties that increase their migratory and invasive capabilities [4–9]. The TGF-β signaling pathway is a major inducer of EMT, primarily through regulation of EMT-related transcription factors [10–15]. SOX4, an oncogenic transcription factor, has been shown to facilitate EMT and tumor progression in various cancers [16–21]. However, its role in TGF-β-induced EMT in LUAD remains unclear.

Emerging studies have revealed the critical role of ADAMTSs in tumorigenesis and metastasis. For example, ADAMTS12 was found to be a prognostic predictor and regulator of EMT in pancreatic cancer [22], whereas ADAMTS8 promoted tumor growth and migration in papillary thyroid cancer by activating the EGFR and MEK/ERK pathways [23]. ADAMTS18, on the other hand, is a tumor suppressor in lung cancer [24]. These findings highlight the crucial but complex roles of ADAMTS proteins in tumor development. ADAMTS16, a critical member of the ADAMTS family, has been reported to facilitate the oncogenesis of gastric cancer by activating the NF-κB pathway [25]. ADAMTS16 was also associated with sensitivity to platinum-based chemotherapy in ovarian cancer cells and prognosis in renal cancer [26, 27]. We found that ADAMTS16 was differentially expressed in multiple LUAD datasets and was the only differentially expressed gene in the ADAMTS family that was associated with overall survival through analysis of public LUAD datasets.

On the basis of the results of bioinformatic analyses and previous studies, we hypothesized that ADAMTS16 is a key regulator of EMT and metastasis in LUAD. This study aimed to evaluate the expression of ADAMTS16 in LUAD, determine its prognostic significance, and explore its role in EMT and metastasis through comprehensive analyses of clinical samples and in vitro and in vivo experiments.

## Materials and methods

### Study design

The goal of this study was to understand the role of ADAMTS16 in EMT and metastasis and explore the regulatory mechanism of ADAMTS16 in LUAD. The expression of ADAMTS16 in public datasets and clinical samples was analyzed to confirm the differential expression and prognostic value of ADAMTS16 in LUAD. Studies involving human samples were performed under the supervision of Institutional Review Board (TJ-IRB20210222). The role and regulatory mechanism of ADAMTS16 were demonstrated via pharmacological inhibitors and genetic inhibition or overexpression approaches. The sample sizes of in vivo experiments were determined on the basis of prior studies and the experience of this animal model, and the mice were allocated randomly to each group. No blinding method was used. All the animal experimental protocols received approval and supervision from the Ethics Committee of the Institutional Animal Care and Use Committee of Tongji Medical College, Huazhong University of Science and Technology (3509). Three biological replicates were performed for in vitro experiments. The sample sizes of all the experiments are indicated in the figure legends. No data were excluded from the analysis.

### Genome-wide association study (GWAS) data source and two-sample Mendelian randomization (MR) analysis

We obtained summary statistics on genetic associations with plasma proteins from previous proteomic studies [28–34] (Supplementary Table 1). Genetic instruments were selected from the protein quantitative trait loci (pQTLs) identified in these studies. We performed MR analysis using the “TwoSampleMR” package [35], employing the Wald ratio and inverse-variance weighted (IVW) methods to estimate MR effects (Supplementary Table 2). Detailed procedures are described in the Supplementary Methods.

### Cell culture and transfection

All the cell lines used in this study were purchased from the American Type Culture Collection (ATCC; Manassas, USA). All the cells were grown at 37 °C in 5% CO2. The sequences of the siRNAs (General Biol, Anhui, China) used in this study are shown in Supplementary Table 3. sh-ADAMTS16 and sh-NC lentiviruses were purchased from JTSBIO (Wuhan, China), and the shRNA sequences used are listed in Supplementary Table 4. The detailed methods are described in the Supplementary Methods.

### Clinical samples

Lung carcinoma and para-carcinoma lung tissues (n = 12) were collected from patients who provided written informed consent and underwent surgical resection at the Thoracic Surgery Department of Wuhan Tongji Hospital between March 2021 and August 2021. The clinical characteristics of the patients are listed in Supplementary Table 5. Peripheral whole blood was collected from patients diagnosed with LUAD and subjected to centrifugation at 2500 rpm/h for 20 min to obtain supernatants. The tissue microarray of LUAD was purchased from Shanghai Outdo Biotech Company (Shanghai, China). ImageJ software was used to calculate the immunohistochemistry (IHC) score of each sample on the basis of the staining intensity and area. The clinical characteristics of the tissue microarray are provided in Supplementary Table 6.

### Western blot (WB)

Western blot assays were conducted as previously described [28]. Briefly, after separation by SDS–PAGE, the proteins were transferred to polyvinylidene fluoride (PVDF) membranes, which were subsequently incubated with primary antibodies overnight at 4°C and then with secondary antibodies for 60 min at RT. The antibodies used in this study are listed in the Supplementary Methods.

### Quantitative real-time PCR

Quantitative real-time PCR was conducted as previously described [36]. Briefly, qRT-PCR analysis was performed via a ChamQ Universal SYBR qPCR Master Mix Kit (Vazyme, Nanjing, China). The mRNA levels were calculated via the 2 − ΔΔCT method, with GAPDH serving as a reference for normalization. The primers used for qRT-PCR are listed in Supplementary Table 7.

### Wound healing and Transwell assays

Wound-healing and Transwell assays were conducted as previously described [28].

The cell migration distances in the wound-healing assay and the number of cells migrating through the transwell inserts in the transwell assay were determined via ImageJ software.

### ChIP-qPCR assays

ChIP assays were performed to elucidate interactions between transcription factors and the promoters of ADAMTS16 and SOX4 via a ChIP-IT Express Enzymatic Kit (Active Motif, 53009). Following fixation with 1% formaldehyde, A549 and H1975 cells were lysed and enzymatically sheared to obtain protein/DNA complexes. These complexes were then immunoprecipitated with antibodies against SOX4 and Smad3 or with IgG. ChIP-enriched DNA fragments were purified and subsequently quantified via qRT‒PCR. The sequences of the primers used for ChIP-qPCR are provided in Supplementary Table 8.

### Immunohistochemistry and immunofluorescence (IF)

The detailed procedures and methods are described in the Supplementary Methods.

### In vivo experiments

Five-week-old male BALB/c nude mice were injected with A549 cells (2 × 10^6 in 100 μl of PBS) via the tail vein to establish lung metastasis model. ADAMT16 was stably silenced to evaluate the effect of ADAMTS16 on metastasis (n = 8 for each group). To assess the effect of the TGF-β1/SOX4 axis on lung metastasis, SOX4 was overexpressed, and SB431542 (HY-10431, MedChemExpress, Shanghai, China) was administered intraperitoneally three times a week for 4 weeks at a concentration of 10 mg/kg (n = 5 for each group). The mice were sacrificed at 6 weeks postinjection, and the whole lungs were removed. The bioluminescence signals of the lungs and chest wall were captured and calculated via an in vivo imaging system.

### Statistical analysis

The statistical analyses were completed with R software (version 4.1.0) and GraphPad 8.0. A normal distribution test was conducted. Normally distributed variables were assessed for differences between two groups via t tests, whereas differences in skewed variables were evaluated via the Mann–Whitney U test. Associations between gene expression levels were evaluated via Pearson’s correlation analysis. *P* < 0.05 was considered to indicate statistical significance.

## Results

### ADAMTS16 is upregulated in LUAD and associated with poor prognosis

**Fig. 1:**
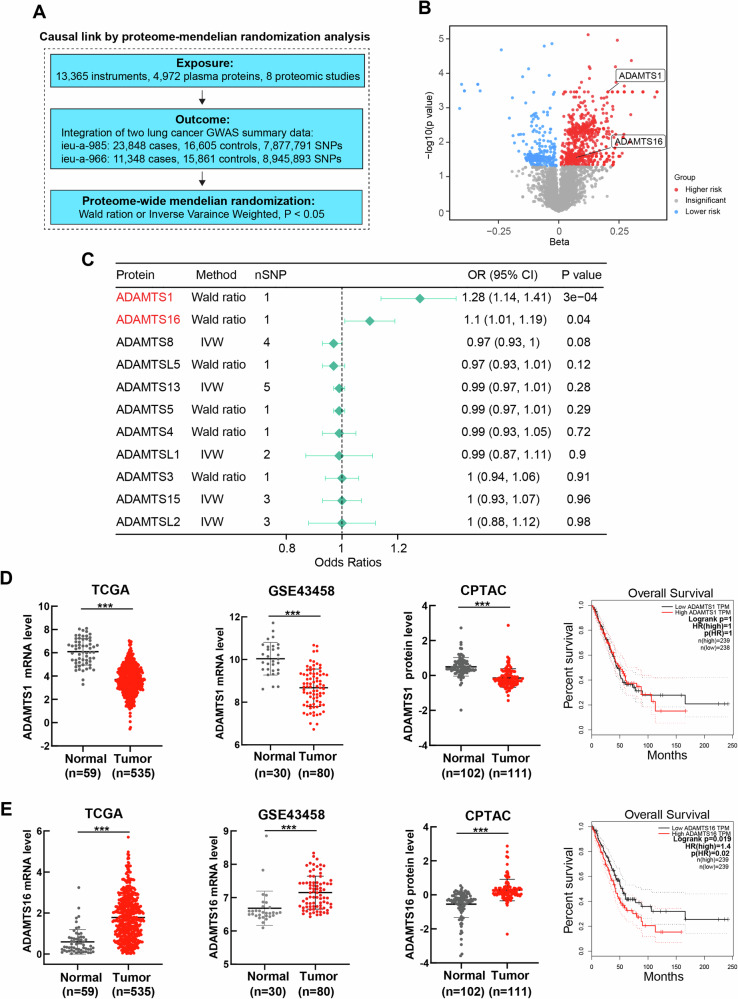
ADAMTS16 was a predictive biomarker of LUAD. **A** Flowchart of proteome-MR analysis. **B** Volcano plot of the results from the proteome-MR analysis showing significant ADAMTS proteins (P < 0.05). **C** Causal effects of proteome-MR analysis between ADAMTS proteins and lung cancer. **D** Differential expression of ADAMTS1 between tumor and normal tissues and survival curves of the high- and low-ADAMTS1 groups in TCGA-LUAD dataset. **E** Differential expression of ADAMTS16 between tumor and normal tissues and survival curves of the high- and low-ADAMTS16 groups in TCGA-LUAD dataset. Gene expression was compared using Mann–Whitney U test, and survival probability was compared via the log-rank test. *P < 0.05; **P < 0.01; ***P < 0.001; ns, not significant.

Mendelian randomization (MR) analysis uses GWAS data to exploit the inherent properties of common genetic variations for a modifiable environmental exposure of interest and has become an important and reliable approach for causal inference [29–31]. To investigate the causal link between ADAMTSs protein expression and lung cancer, proteome-wide two-sample MR analysis was conducted. We collected the most recent large-scale plasma proteome studies with sample sizes of over 1000 (Supplementary Table 1). After integration, we obtained around 5,000 unique proteins, each with its corresponding pQTL instruments (Fig. 1A). Two large lung cancer GWAS with 23,848 cases, 16,605 controls and 11,348 cases, 15,861 controls respectively were included and meta-integrated. MR analysis was performed using these proteins with highly related SNPs as exposures and the risk of lung cancer as the outcome (Fig. 1A). MR analysis via the Wald ratio or IVW method revealed a causal relationship between ADAMTS1 or AMAMTS16 and the risk of lung cancer (Fig. 1B, Supplementary Table 2). Higher levels of ADAMTS1 (OR: 1.28, 95% CI: 1.14–1.41) and ADAMTS16 (OR: 1.1, 95% CI: 1.01--1.19) were genetically predicted to be associated with lung cancer (Fig. 1C).

**Fig. 2:**
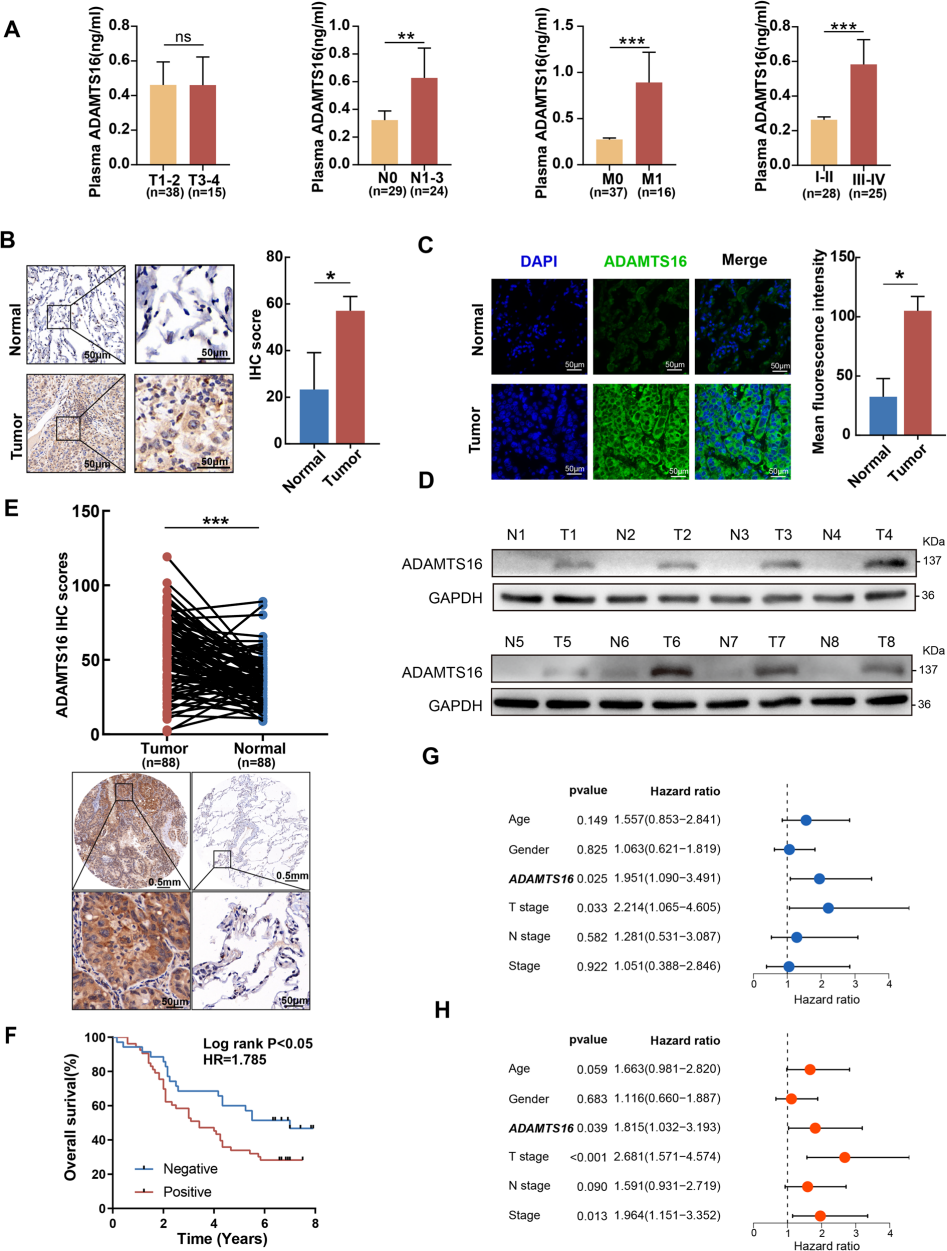
ADAMTS16 was upregulated and predicted poor survival in LUAD patients. **A** Plasma ADAMTS16 levels in patients with LUAD (n = 53). **B–D** Comparison of the protein levels of ADAMTS16 between LUAD and normal lung tissue samples via western blotting, IHC, and IF. **E** IHC score of ADAMTS16 in tissue microarray samples (n = 88). **F** Survival curves of patients with positive or negative ADAMTS16 expression in the tissue microarray. **G**, **H** Univariate and multivariate Cox regression analyses indicating the effect of the IHC score of ADAMTS16 on survival in the tissue microarray cohort. The IHC scores and relative immunofluorescence intensities were expressed as the means ± SEMs and compared via the Mann‒Whitney U test. Survival probability was compared with the log-rank test. *P < 0.05; **P < 0.01; ***P < 0.001; ns, not significant.

We then explored the expression profiles of ADAMTS1 and ADAMTS16 in multiple LUAD cohorts. Although both ADAMTS1 and ADAMTS16 were differentially expressed between LUAD and adjacent lung tissues in multiple datasets, only ADAMTS16 was associated with overall survival in the TCGA cohort (Fig. 1D, E). Therefore, ADAMTS16 may play a crucial role in LUAD tumor development. High plasma ADAMTS16 levels were associated with node and distant metastasis (Fig. 2A, Table 1). Clinical specimens confirmed the upregulation of ADAMTS16 in LUAD (Fig. 2B–D, Supplementary Table 5). The IHC score of LUAD tissue in the tissue microarray was greater than that of normal lung tissue (Fig. 2E, Supplementary Table 6). Kaplan–Meier survival analysis of the tissue microarray cohort showed that patients with negative ADAMTS16 immunostaining survived longer than those with positive ADAMTS16 expression (Fig. 2F). Univariate and multivariate Cox regression analyses of the tissue microarray data revealed that the expression of ADAMTS16 predicted poor prognosis in LUAD patients (Fig. 2G, H).

**Table 1:** Clinical features of LUAD patients.

Variables	Total	ADAMTS16-High	ADAMTS16-Low	*P* value
Age				
<65	41 (77.36%)	19 (70.37%)	22 (84.62%)	0.3626
≥65	12 (22.64%)	8 (29.63%)	4 (15.38%)	
Sex				
Female	27 (50.94%)	13 (48.15%)	14 (53.85%)	0.8887
Male	26 (49.06%)	14 (51.85%)	12 (46.15%)	
*T*				
1–2	38 (71.7%)	18 (66.67%)	20 (76.92%)	0.6005
3–4	15 (28.3%)	9 (33.33%)	6 (23.08%)	
*N*				
0	29 (54.72%)	10 (37.04%)	19 (73.08%)	0.0183
1–3	24 (45.28%)	17 (62.96%)	7 (26.92%)	
*M*				
0	37 (69.81%)	15 (55.56%)	22 (84.62%)	0.045
1	16 (30.19%)	12 (44.44%)	4 (15.38%)	
Stage				
I–II	28 (52.83%)	9 (33.33%)	19 (73.08%)	0.0087
III–IV	25 (47.17%)	18 (66.67%)	7 (26.92%)	

### ADAMTS16 promotes EMT and migration of LUAD cells

**Fig. 3:**
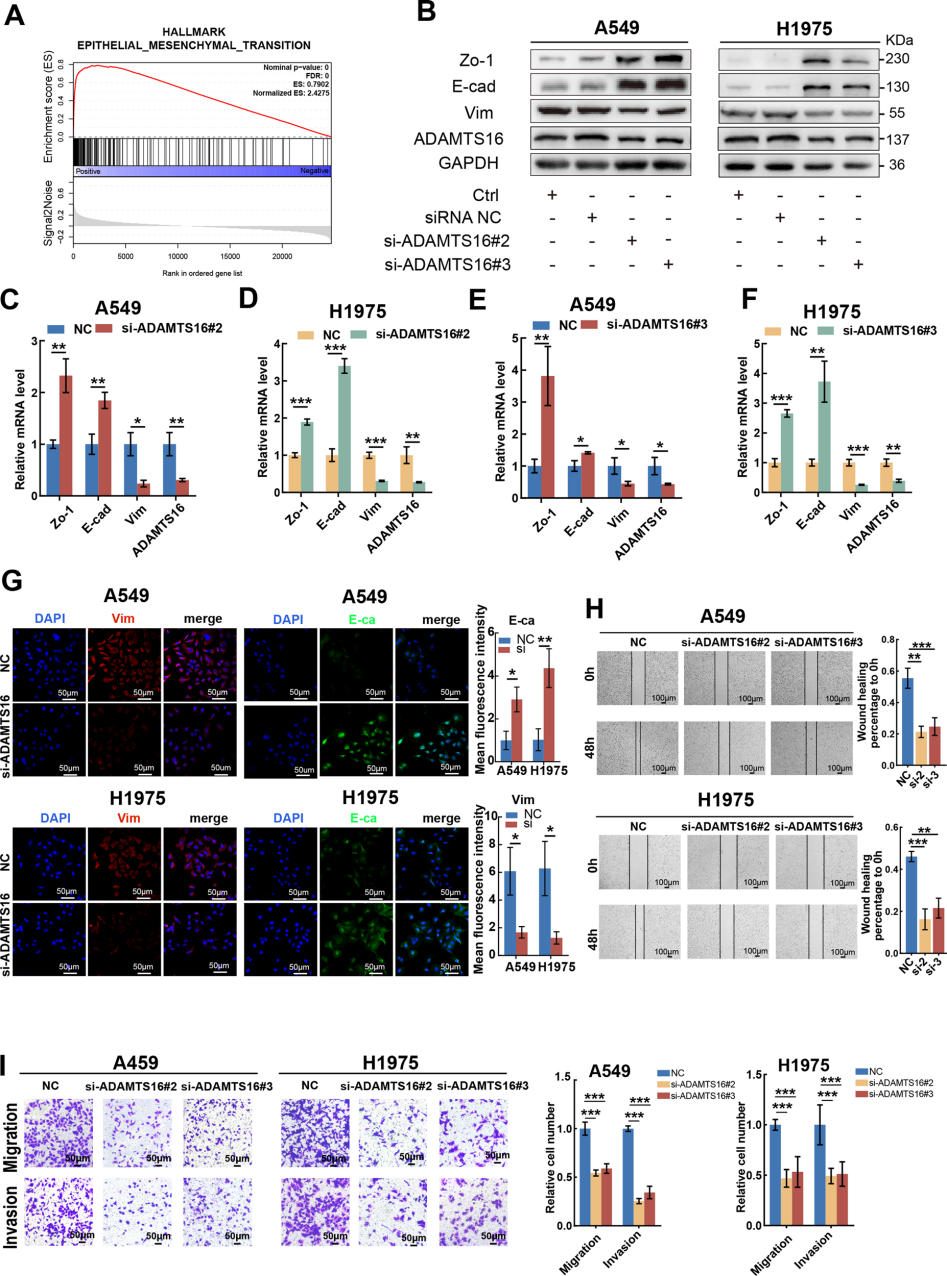
Silencing ADAMTS16 impeded EMT in LUAD. **A** GSEA plot showing the association between ADAMTS16 and EMT. **B** Changes in the expression of EMT-related markers indicated by immunoblotting after the knockdown of ADAMTS16. **C**–**F** Changes in the expression of EMT-related markers indicated by qRT-PCR after ADAMTS16 was silenced. **G** Changes in expression of EMT-related marker indicated by IF after knockdown of ADAMTS16. **H** Wound healing assay showing the change in the migration ability of LUAD cells after the knockdown of ADAMTS16. **I** Transwell assay showing the changes in the migration and invasion abilities of LUAD cells after silencing ADAMTS16. Data was expressed as mean ± SEM (n = 3 for each group) and was compared via Student’s t test. *P < 0.05; **P < 0.01; ***P < 0.001; ns, not significant.

To elucidate the underlying mechanism of ADAMTS16 in LUAD, we performed GSEA in the TCGA-LUAD cohort, which revealed a positive correlation between ADAMTS16 and EMT (Fig. 3A). The results of GSEA also demonstrated that ADAMTS16 was associated with the structural constituents and metabolic processes of the ECM (Supplementary Fig. 1). ADAMTS16 was silenced, and the silencing efficiency was evaluated in A549 and H1975 cells (Supplementary Fig. 2). PCR, WB, and IF analyses revealed increased expression of ZO-1 and E-cadherin and decreased expression of vimentin after ADAMTS16 knockdown, indicating that ADAMTS16 inhibition impaired EMT in LUAD cells (Fig. 3B–G). Additionally, as shown by the wound healing and transwell assays, silencing ADAMTS16 impeded the migratory and invasive potential of A549 and H1975 cells (Fig. 3H, I). These findings suggest that ADAMTS16 is a driver gene of EMT.

**Fig. 4:**
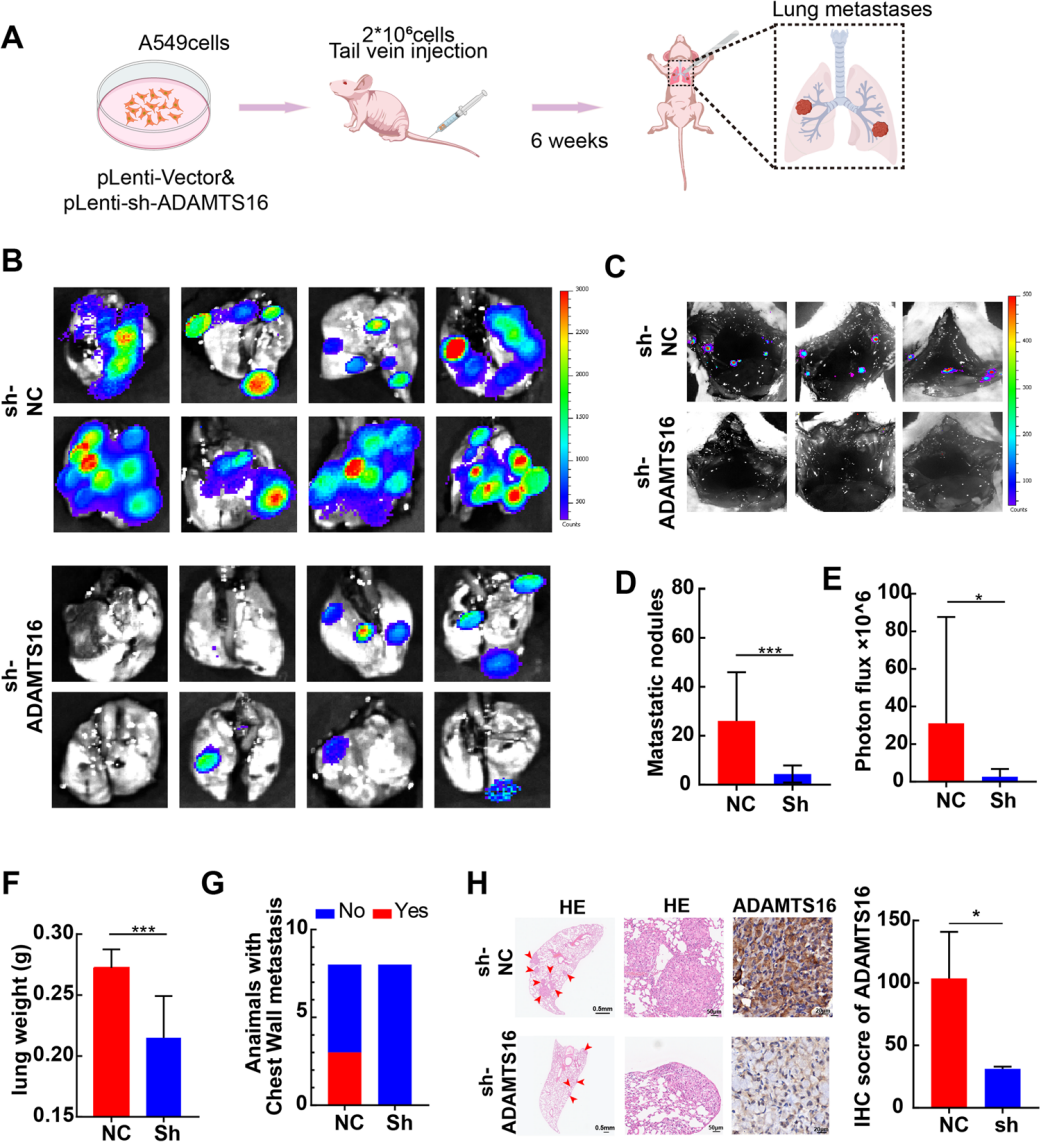
Inhibition of ADAMTS16 repressed metastasis of LUAD in vivo. **A** Schematic flowchart presenting the procedure for generating the in vivo metastasis model. **B** Metastatic lesions in the lungs of the control and ADAMTS16-silenced groups captured by bioluminescence imaging. **C** Metastatic lesions in the chest wall of the control and ADAMTS16-silenced groups captured by bioluminescence imaging. **D** Counts of metastatic nodules on the surface of the lungs in the control and ADAMTS16-silenced groups. **E** Luciferase activity in the lungs of the control and ADAMTS16-silenced groups. **F** Lung weight in the control and ADAMTS16-silenced groups. **G** Distribution of chest wall metastases in the control and ADAMTS16-silenced groups. **H** H&E staining and IHC staining of ADAMTS16 in lung metastatic lesions from the control and ADAMTS16-silenced groups. Data was expressed as the means ± SEM (n = 8 for each group) and was compared via Student’s t test. *P < 0.05; **P < 0.01; ***P < 0.001; ns, not significant.

To further evaluate the effect of ADAMTS16 on the metastasis of LUAD, nude mice received tail vein injections of A549 cells with stable ADAMTS16 knockdown or control A549 cells (Fig. 4A). The expression of ADAMTS16 validated the stable knockdown of ADAMTS16 in A549 cells, and sh-ADAMTS16#2 was selected for further animal models (Supplementary Fig. 3). Nude mice were sacrificed at 6 weeks after tail vein injection to evaluate metastatic lesions in the lung and chest wall. ADAMTS16 knockdown significantly reduced metastatic nodules in the lungs (Fig. 4B, D). The weight and luciferase activity of the lungs are decreased in the ADAMTS16-knockdown group (Fig. 4E, F). Notably, in contrast to those in the control group, in which three mice developed chest wall metastasis, no mice with ADAMTS16 knockdown developed chest wall metastasis (Fig. 4C, G). IHC staining of tumor tissues further confirmed the inhibition of ADAMTS16 in the ADAMTS16-knockdown group (Fig. 4H).

### ADAMTS16 promotes EMT by facilitating the activation of TGF-β1

**Fig. 5:**
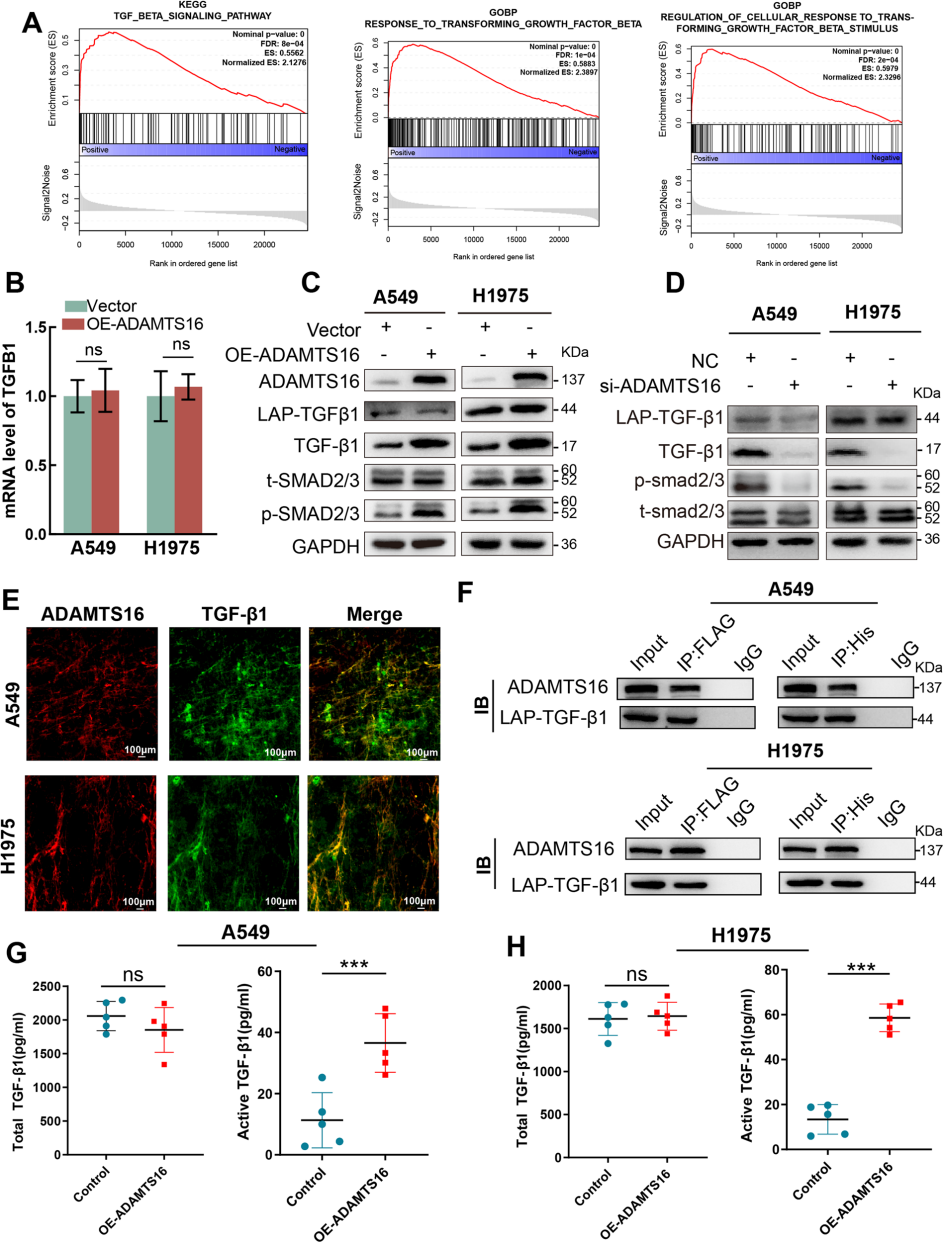
ADAMTS16 promoted the activation of TGF-β1. **A** GSEA plot showing the association between ADAMTS16 and the TGF-β signaling pathway. **B** Effect of ADAMTS16 overexpression on transcription of TGFB1. **C** Immunoblots showing the impact of ADAMTS16 overexpression on the activity of the TGF-β signaling pathway. **D** Immunoblots showing the impact of ADAMTS16 knockdown on the activity of the TGF-β signaling pathway. **E** IF showing co-localization of ADAMTS16 and TGF-β1 in the ECM. **F** Co-IP showing the interaction between ADAMTS16 and LAP-TGF-β1. **G**, **H** The protein levels of active and total TGF-β1 in the cell supernatants of LUAD cells with or without ADAMTS16 overexpression. Data was expressed as mean ± SEM (n = 3 for each group) and was compared via Student’s t test. *P < 0.05; **P < 0.01; ***P < 0.001; ns, not significant.

GSEA of TCGA-LUAD data indicated that ADAMTS16 was positively related to the TGF-β signaling pathway (Fig. 5A). However, ADAMTS16 overexpression had no effect on LAP-TGF-β1 but increased mature TGF-β1 and activated Smad2/3 (Fig. 5B–C). Immunofluorescence of LUAD cells indicated that ADAMTS16 was co-localized with TGF-β1 in the ECM (Fig. 5E). Co-IP revealed the interaction between ADAMTS16 and LAP-TGF-β1 (Fig. 5F). Silencing ADAMTS16 decreased mature TGF-β1 and inactivated Smad2/3 (Fig. 5D). Overexpression of ADAMTS16 increased active TGF-β1 but not total TGF-β1 in the cell supernatants of A549 and H1975 cells (Fig. 5G, H).

**Fig. 6:**
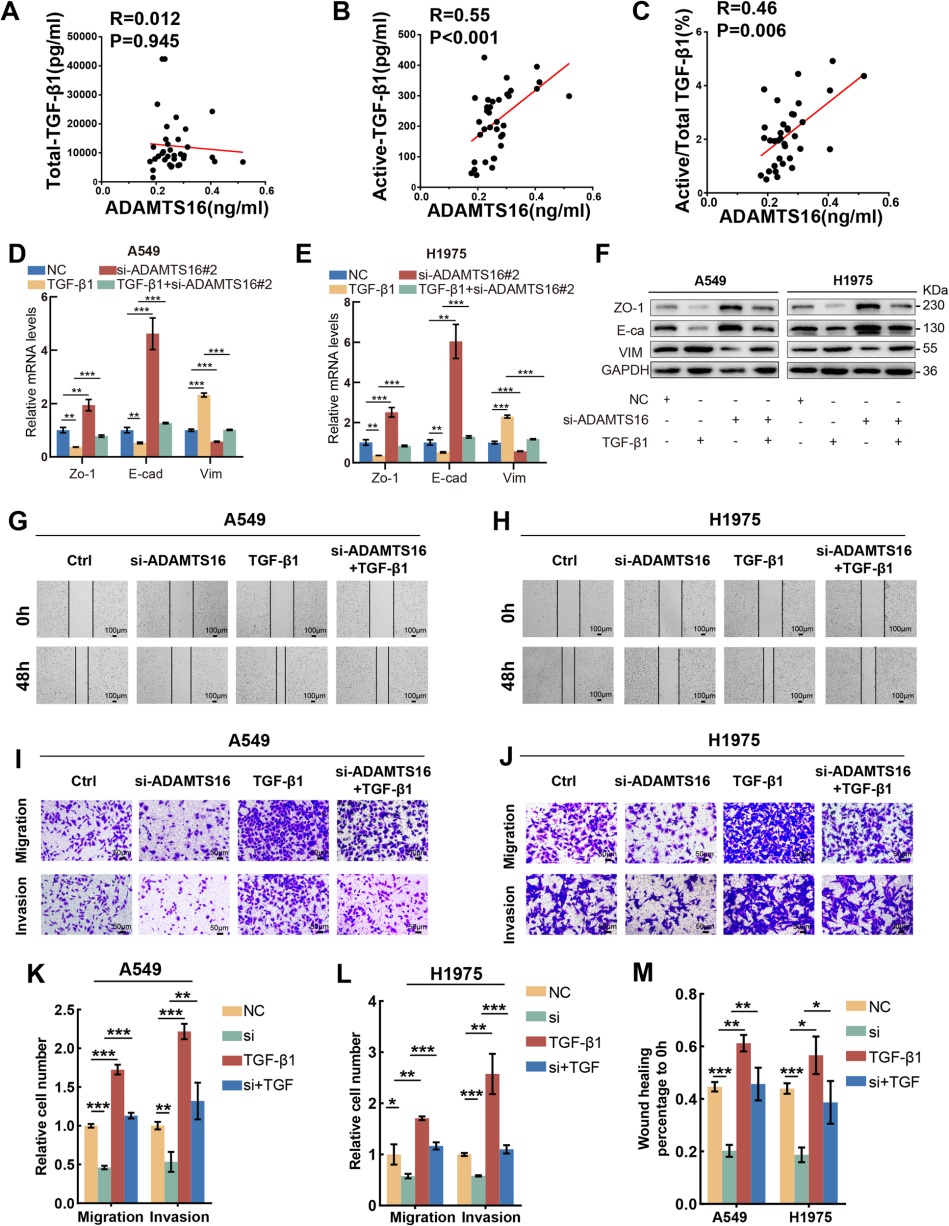
ADAMTS16 regulated EMT via TGF-β1. **A**–**C** Correlations between plasma ADAMTS16 and active or total TGF-β1 in patients with LUAD. **D**, **E** Effects of treatment with TGF-β1 or si-ADAMTS16 on mRNA expression of EMT-related biomarkers indicated by qRT-PCR. **F** Immunoblots showing the effects of treatment with TGF-β1 or si-ADAMTS16 on the protein expression of EMT-related biomarkers. **G**, **H**, **M** Wound healing assay showing the effects of treatment with TGF-β1 or si-ADAMTS16 on the migration ability of LUAD cells. **I**, **J**, **K**, **L** Transwell assay showing the effects of treatment with TGF-β1 or si-ADAMTS16 on the migration and invasion ability of LUAD cells. **A** Data was analyzed by Pearson correlation analysis (n = 35). **D**, **E**, **K**–**M** Data was expressed as mean ± SEM (n = 3 for each group) and compared using Student’s t-test. *P < 0.05; **P < 0.01; ***P < 0.001; ns, not significant.

The results of ELISA suggested that plasma ADAMTS16 was positively correlated with the active TGF-β1 and the percentage of active TGF-β1 but not total TGF-β1 in patients with LUAD (Fig. 6A–C). We also explored the relationship between ADAMTS16 and TGF-β receptors. The results of IP assay did not indicate an interaction between ADAMTS16 and TGF-βRI or TGF-βRII (Supplementary Fig. 4). Collectively, these findings demonstrated that ADAMTS16 interacted with LAP-TGF-β1 and promoted the maturation of TGF-β1 to activate the TGF-β signaling pathway. The TGF-β signaling pathway is the major inducer of EMT. Silencing ADAMTS16 repressed EMT, whereas recombinant TGF-β1 protein attenuated this repressive effect of ADAMTS16 knockdown on EMT (Fig. 6D–F). Treatment with recombinant TGF-β1 protein also restored the impaired migration and invasion abilities of LUAD cells caused by ADAMTS16 knockdown (Fig. 6G–M). Therefore, ADAMTS16 induces EMT via the TGF-β signaling pathway.

### SOX4 activates the transcription of ADAMTS16

**Fig. 7:**
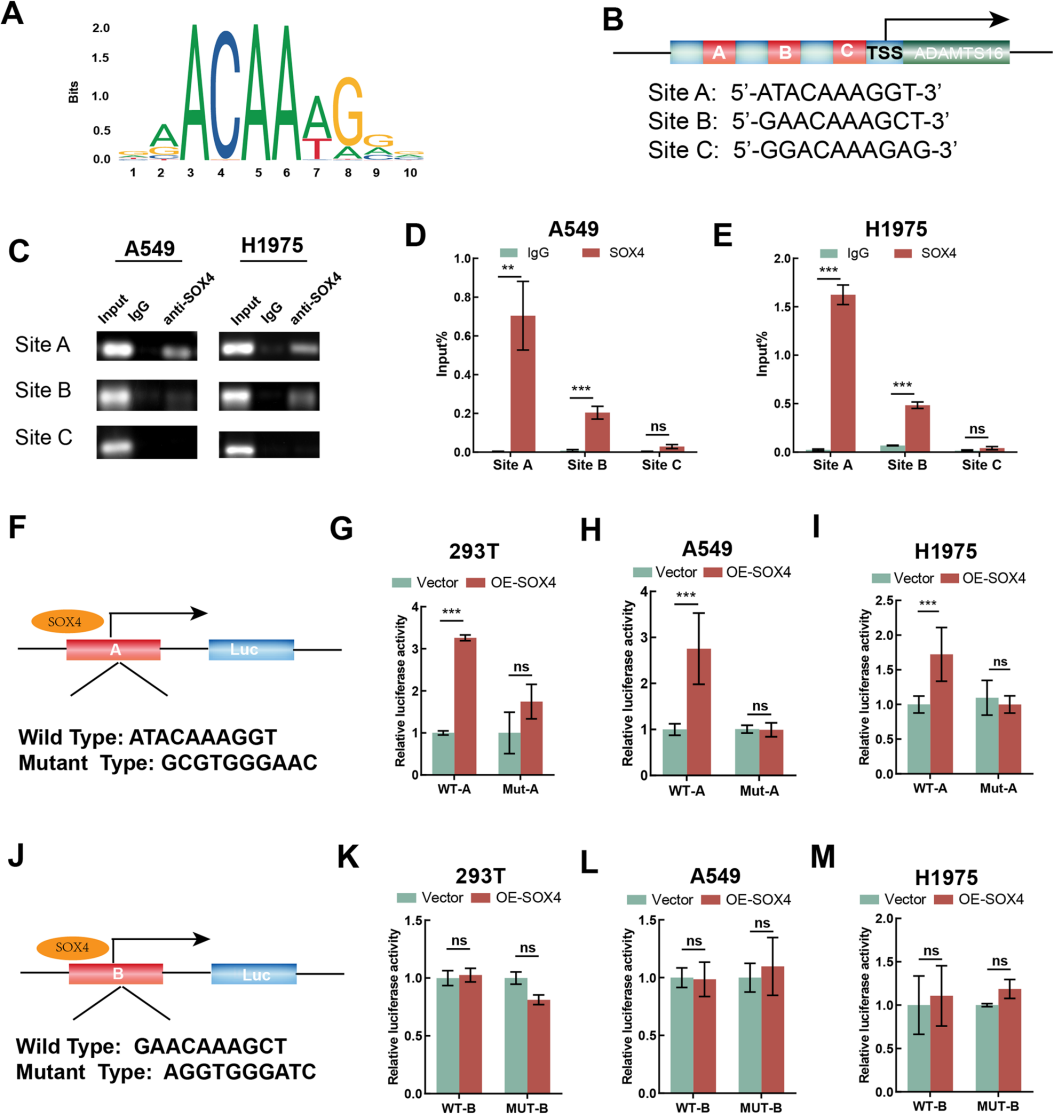
SOX4 activates transcription of ADAMTS16. **A** The SOX4-binding motif of ADAMTS16 acquired from JASPAR. **B** Schematic diagram displaying the predicted binding sites in the promoter of ADAMTS16. **C** ChIP assay indicating the occupancy of SOX4 at the promoter of ADAMTS16. **D**, **E** ChIP-qPCR results indicating the interaction between SOX4 and the promoter of ADAMTS16. **F** Schematic drawing of luciferase reporter plasmids containing the wild-type or mutated SOX4-binding sequence (site A) in the promoter of ADAMTS16. **G**–**I** Dual-luciferase reporter assay indicating the luciferase activity of the reporter plasmid containing the wild-type or mutated binding sequence (site A) of the ADAMTS16 promoter. **J** Schematic drawing of luciferase reporter plasmids containing the wild-type or mutated SOX4-binding sequence (site B) of the ADAMTS16 promoter. **K**‒**M** Dual-luciferase reporter assay indicating the luciferase activity of reporter plasmids containing the wild-type or mutated sequence (site B) of the ADAMTS16 promoter. Data was expressed as mean ± SEM (n = 3 for each group) and was compared using Student’s t-test. *P < 0.05; **P < 0.01; ***P < 0.001; ns, not significant.

SOX4 manipulates the expression of a subset of genes to drive tumorigenesis and metastasis [32–34]. Correlation analyses revealed a positive relationship between the expression of ADAMTS16 and SOX4 in both the TCGA and GSE43458 datasets (Supplementary Fig. 5A, B). Moreover, SOX4 is upregulated in LUAD in public datasets and clinical samples (Supplementary Fig. 6A–E). We then acquired the sequences in the promoter region of ADAMTS16 that are most likely to bind with SOX4 via the JASPAR website (Fig. 7A, B). The results of ChIP-qPCR showed an occupancy of SOX4 at site A and site B, with a higher binding rate observed at site A (Fig. 7C–E). The results of dual-luciferase reporter assay suggested that overexpression of SOX4 increased the transcriptional activity of the reporter plasmid containing the wild-type site A of the ADAMTS16 promoter, whereas the effect of SOX4 was abolished by the mutation of site A (Fig. 7F–I). However, the transcriptional activity of site B was not affected by SOX4 overexpression (Fig. 7J–M). These findings collectively support the conclusion that SOX4 facilitates the transcription of ADAMTS16.

### TGF-β regulates expression of ADAMTS16 via SOX4

**Fig. 8:**
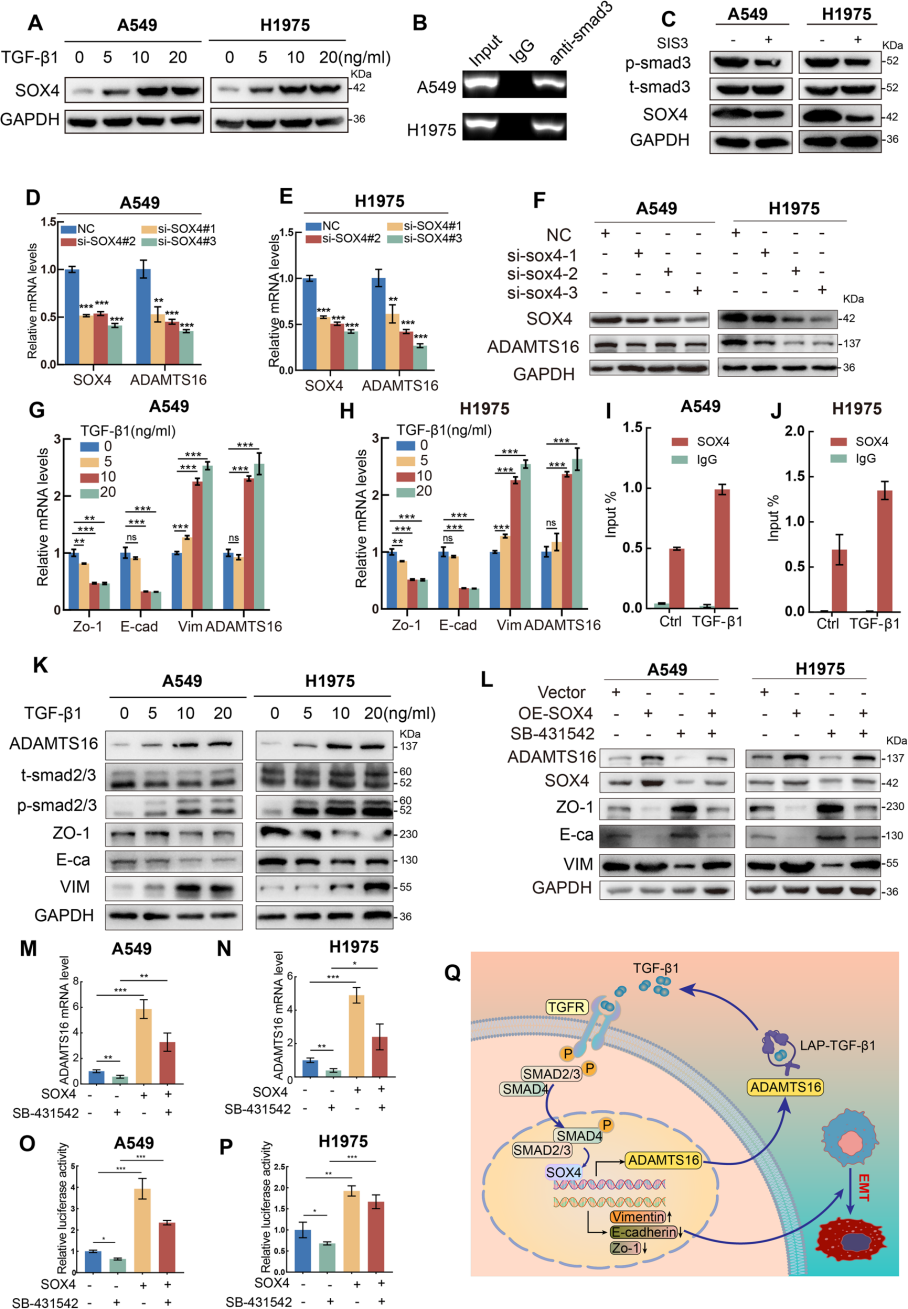
The TGF-β/SOX4 axis controls the expression of ADAMTS16. **A** Effect of TGF-β1 stimulation on protein expression of SOX4. **B** ChIP assay indicating the occupancy of Smad3 at the promoter of SOX4. **C** Effect of SIS3 on protein expression of SOX4. **D**, **E** The impact of silencing SOX4 on the mRNA levels of ADAMTS16 and SOX4. **F** Immunoblots demonstrating the impact of silencing SOX4 on the protein levels of ADAMTS16 and SOX4. **G**, **H** Effect of TGF-β1 stimulation on mRNA expression of EMT-related biomarkers and ADAMTS16. **I**, **J** Effect of TGF-β1 on binding of SOX4 to the promoter of ADAMTS16. **K** Effect of TGF-β1 stimulation on protein expression of EMT-related biomarkers and ADAMTS16. **L** Immunoblots showing the effects of SB-431542 treatment or SOX4 overexpression on the protein expression of SOX4, ADAMTS16, and EMT-related biomarkers. **M**, **N** The effects of SB-431542 treatment or SOX4 overexpression on the mRNA expression of ADAMTS16**. O**, **P** Dual-luciferase reporter assay indicating the effects of SB-431542 treatment or SOX4 overexpression on the transcriptional activity of ADAMTS16. **Q** Schematic of the working model. Data was expressed as mean ± SEM (n = 3 for each group) and was compared using Student’s t-test. *P < 0.05; **P < 0.01; ***P < 0.001;ns, not significant.

SOX4 has been proven to be required for a subset of processes triggered by TGF-β1 [18, 34–36]. We found that TGF-β1 promoted expression of SOX4 in a dose-dependent manner (Fig. 8A, Supplementary Fig. 7A). Next, we explored how TGF-β1 regulated expression of SOX4. Smad3 acts as a critical transcription factor of the TGF-β signaling pathway. We obtained the sequence in the promoter of SOX4 that Smad3 might bind to (Supplementary Fig. 7B). The results of ChIP-qPCR indicated an occupancy of Smad3 on the promoter of SOX4 (Fig. 8B, Supplementary Fig. 7C). Additionally, SIS3, an inhibitor of Smad3, suppressed the expression of SOX4 (Fig. 8C, Supplementary Fig. 7D-E). These results suggested that TGF-β1 regulated the expression of SOX4 through the transcription factor SMAD3. Suppression of SOX4 inhibited the expression of ADAMTS16 (Fig. 8D–F). Treatment with TGF-β1 also increased ADAMTS16 expression in a dose-dependent manner (Fig. 8G, H, K). TGF-β1 promoted the binding of SOX4 to the promoter of ADAMTS16 (Fig. 8I, J, Supplementary Fig. 7F). SB431542, a TGFBR1 inhibitor, suppressed EMT and the expression of ADAMTS16, which was rescued by SOX4 overexpression (Fig. 8L). A dual-luciferase reporter assay and qRT-PCR revealed that SB431542 reduced the transcriptional activity of ADAMTS16, but SOX4 overexpression counteracted this inhibitory effect (Fig. 8M‒P). An animal model revealed that inhibition of the TGF-β signaling pathway by SB431542 reduced lung metastasis and the expression of ADAMTS16, whereas overexpression of SOX4 facilitated lung metastasis and the expression of ADAMTS16 (Supplementary Fig. 8A–G). The overexpression of SOX4 eliminated the inhibitory effect of SB431542 on lung metastasis. These findings collectively support that TGF-β1 regulates the expression of ADAMTS16 via SOX4 and that ADAMTS16 forms a positive feedback loop with TGF-β/SOX4 axis to drive EMT and metastasis (Fig. 8Q).

## Discussion

ADAMTSs have been identified as critical regulators of malignant behaviors such as angiogenesis and EMT [22, 37–39]. Nevertheless, the functions of ADAMTSs in LUAD have not been fully characterized [24, 40]. This study identified ADAMTS16 as a key prognostic factor in LUAD, demonstrated that ADAMTS16 promoted EMT and metastasis by activating TGF-β1, and revealed a ADAMTS16/TGF-β1/SOX4 positive feedback loop that drives EMT and metastasis.

In this study, analysis of multiple public datasets and clinical samples suggested that ADAMTS16 was upregulated in LUAD tissues and that the upregulation of ADAMTS16 was associated with metastasis and unfavorable survival in LUAD patients. Our study provides a novel and convenient strategy for predicting outcome of patients with LUAD, which can be achieved by measuring the expression of ADAMTS16. Next, GSEA of the TCGA-LUAD cohort demonstrated that ADAMTS16 expression was positively related to EMT. Other members of the ADAMTS family, such as ADAMTS1, ADAMTS12, and ADAMTS18, are also regulators of EMT [22, 38, 41]. Our in vitro experiments demonstrated that inhibition of ADAMTS16 markedly suppressed EMT and cell migration. Previous studies reported that ADAMTS16 facilitates gastric carcinogenesis and predicts unfavorable outcomes in renal cancer patients [25, 27]. Thus, the oncogenic role of ADAMTS16 may be universal across different malignancies. An in vivo metastasis model suggested that knockdown of ADAMTS16 reduced lung metastasis and pleural dissemination, indicating that targeting ADAMTS16 may be a feasible strategy for preventing invasion and distant metastasis in LUAD patients.

EMT is initiated and regulated by multiple factors, among which TGF-β has been identified as a core regulator [12, 42, 43]. Cancer cells with TGF-β1 signaling pathway inhibited exhibited impaired EMT and metastatic potential [44–48]. TGF-β1 and ADAMTS16 can be secreted by cells to become crucial constituents of ECM. TGF-β1 is sequestered in the ECM in a latent form, which requires activation to perform its biological functions. Members of the ADAMTS family are regulators of the TGF-β signaling pathway. LAP-TGF-β1 and the TGF-β receptor were identified as substrates of ADAMTS2, 3, and 14 [49]. We found that ADAMTS16 facilitated TGF-β-induced EMT via interaction with LAP-TGF-β1 rather than TGF-β receptors. ADAMTS16 has also been reported to interact with LAP-TGF-β to transform it into the active form in cardiac fibroblasts and promote fibrosis [50], suggesting that ADAMTS16 activates LAP-TGF-β to participate in various biological processes. Therefore, activation of TGF-β1 is the major mechanism underlying the effect of ADAMTS16 on the TGF-β signaling pathway. Our findings provide novel insights into the regulatory mechanism of TGF-β-induced EMT.

SOX4 participates in a subset of cellular events that modulate development and regeneration by modulating gene transcription. SOX4 facilitates EMT and metastasis in multiple malignancies [17, 51–54]. Previous studies have also demonstrated that SOX4 is a downstream target of the TGF-β signaling pathway and that SOX4 is a master regulator of TGF-β-induced EMT via the regulation of gene transcription [17, 18, 35, 36]. Our findings suggested that TGF-β promoted the transcription and expression of SOX4 via Smad3. Our study also demonstrated that TGF-β1 stimulated the expression of ADAMTS16 via SOX4 which controlled the transcription of ADAMTS16. In vivo experiments demonstrated that overexpression of SOX4 reversed the inhibitory effect of the TGF-β inhibitor on lung metastasis. Thus, ADAMTS16 forms a positive feedback loop with TGF-β1/ SOX4 axis to drive EMT and metastasis. Although Yao et al. reported the role of ADAMTS16 in the activation of LAP-TGF-β1 in cardiac fibroblasts [50], the interaction between ADAMTS16 and LAP-TGF-β1 in tumor cells and the role of ADAMTS16 in EMT and metastasis in LUAD have not been clarified. Additionally, the effects of TGF-β1 on ADAMTS16 expression and the underlying regulatory mechanism have not been revealed. Our study elucidated the role of ADAMTS16 in EMT and metastasis, indicated how TGF-β1 regulated ADAMTS16 through SOX4, and revealed an ADAMTS16/TGF-β/SOX4 positive feedback loop that drove EMT and metastasis. These findings of our study facilitate the understanding of EMT and metastasis and identify potential therapeutic target for treating metastasis.

Although we revealed the role of ADADMATS16 in LUAD, this study has several limitations. While we verified the prognostic value of ADAMTS16 in LUAD through analyses of public databases and clinical samples, the sample size was insufficient to provide robust evidence for clinical application. Additionally, this study focused on the function of ADAMTS16 in EMT because of its significant positive correlation with EMT. However, ADAMTS16 may also participate in other malignant phenotypes of LUAD, such as cell viability and angiogenesis. Third, this study exclusively investigated the role of ADAMTS16 in LUAD, and its function in other malignancies is unclear.

## Conclusions

This study clarifies the role of ADAMTS16 in the prognosis and metastasis of LUAD patients. This study also reveals an ADAMTS16/TGF-β/SOX4 positive feedback loop that drives EMT and metastasis in LUAD. ADAMTS16 may be a useful biomarker for evaluating the prognosis of patients with LUAD. Furthermore, disrupting the feedback loop between ADAMTS16 and TGF-β may represent a promising therapeutic approach for preventing metastasis in LUAD. This study provides potential prognostic indicators and therapeutic targets for LUAD and sheds new light on the mechanism of EMT and metastasis in LUAD.

## Supplementary information

supplementary methods

Supplementary tables 2

Supplementary figures(1–8) and tables(1,3–8)

full western blot

## Data Availability

The transcriptome datasets of LUAD analyzed in this study are available in the TCGA (https://portal.gdc.cancer.gov/) and GEO (http://www.ncbi.nlm.nih.gov/geo) databases, respectively. The proteome dataset of LUAD analyzed in this study is available in the CPTAC database (https://pdc.cancer.gov/pdc/browse).
