# Supplementary material for: ADAMTS16 drives epithelial-mesenchymal transition and metastasis through a feedback loop upon TGF-β1 activation in lung adenocarcinoma
Source: Cell Death Dis. 2024 Nov 17;15(11):837. doi: 10.1038/s41419-024-07226-z (PMC11570625; doi:10.1038/s41419-024-07226-z)
Supplement: Supplementary file 1 — supplementary methods [file 41419_2024_7226_MOESM1_ESM.docx]

**GWAS data source**

We gathered summary statistics on genetic associations with plasma proteins from previous proteomic studies (**Supplementary Table 1**), using the SOMALogic, Olink, and xMAP platforms. GWAS data for lung cancer were sourced from the European Bioinformatics Institute (ebi-a-985, ebi-a-966) in the GWAS catalog (https://www.ebi.ac.uk/gwas/). All participants provided informed consent, and ethics approvals were granted by the relevant authorities.

**Two-sample MR analysis**

Genetic instruments were selected from protein quantitative trait locis (pQTLs) in the eight proteomic studies. We performed Mendelian randomization (MR) analysis using the "TwoSampleMR" package (35). For each plasma protein with a single instrument, we used the Wald ratio method to estimate the change in log odds of lung cancer per standard deviation increase in protein levels. For proteins with multiple instruments, we applied the inverse-variance weighted (IVW) method (**Supplementary Table 2**).

**Immunohistochemistry (IHC)**

Lung tissues from humans and mice were fixed with 4% formalin for 24 hours and subsequently paraffin embedded, resulting in lung slides (5μm thick). Lung slices were incubated with primary antibodies, including anti-ADAMTS16 (Abmart, PA6250, 1:100), anti-E-cadherin (Abcam, ab40772, 1:500), anti-Vimentin (Abcam, ab92547 1:500), anti-Ki67 (Abcam, ab15580, 1:400), and anti-SOX4 (Santa Cruz, sc-518016, 1:200) at 4°C overnight, followed by incubation with biotinylated secondary antibodies for 30 minutes. The slides were treated with DAB working solution before hematoxylin staining and sealing. IHC score was determined via ImageJ software.

**Immunofluorescence (IF) staining**

After fixation with 4% paraformaldehyde and permeabilization with 0.1% Triton X-100, the cells were stained with antibodies against ADAMTS16 (1:100), E-cadherin (1:500), or vimentin (1:1000) at 4°C overnight and then with Cy3 or FITC-labeled secondary antibodies (Proteintech, SA00009/SA00003, 1:200) for 1 hour. The cell nuclei were stained with DAPI for 6 minutes. Formalin-fixed paraffin-embedded tissues were used for immunofluorescence staining. Immunofluorescence intensity was quantified using ImageJ software.

**Plasmid and siRNA transfection**

Cells were transfected with pcDNA3.1-Flag-ADAMTS16, pcDNA3.1-SOX4, or pcDNA3.1-His-TGFB1 plasmids (Genecreate, Wuhan, China) using Lipofectamine 3000 (Invitrogen) to overexpress ADAMTS16, LAP-TGF-β, or SOX4, respectively. LUAD cells were transiently transfected with si-ADAMTS16 and si-SOX4 (General Biol, Anhui, China) using Lipofectamine 2000 (Invitrogen).

**Lentivirus transduction and establishment of LUAD cell lines with stable ADAMTS16 suppression**

Sh-ADAMTS16 and sh-NC lentiviruses were purchased from JTSBIO (Wuhan, China), and the shRNA sequences used are listed in **Supplementary Table 7**. LUAD cell lines were transfected with lentivirus for 24 h using 5 µg/ml polybrene (JTSBIO, Wuhan, China). Cells with ADAMTS16 stably suppressed were selected with 2 μg/ml puromycin (JTSBIO, Wuhan, China).

**Antibodies used in WB**

ADAMTS16 (Abmart, PA6250, 1:500), vimentin (Proteintech, 10366-1-AP, 1:1000), E-cadherin (CST, 3195, 1:1000), Flag (Abmart, [M20008](http://www.ab-mart.com.cn/page.aspx?node=%2059%20&id=%20968), 1:100), His (Abmart, [M20001](http://www.ab-mart.com.cn/page.aspx?node=%2059%20&id=%20959), 1:100), ZO-1 (Proteintech, 21773-1-AP, 1:1000), TGF-β1 (Proteintech 21898-1-AP, 1:1000), t-smad2/3 (CST, 8685S, 1:1000), p-smad2/3 (CST, 8828S, 1:1000), SOX4 (Santa Cruz, sc-518016, 1:500), and GAPDH (Proteintech 60004-1-Ig, 1:5000), TGF-βRI (UpingBio Technology, YP-Ab-13695, 1:1000), TGF-βRII (UpingBio Technology, YP-Ab-13696, 1:1000), t-smad3 (CST, 9523, 1:1000), p-smad3 (abcam, 52903, 1:1000).

**Dual-luciferase reporter assay**

Various wild-type or mutated ADAMTS16 promoter fragments were subcloned and inserted into the pGL3-basic vector to construct pGL3-ADAMTS16 WT1-Luc, pGL3-ADAMTS16 MUT1-Luc, pGL3-ADAMTS16 WT2-Luc, and pGL3-ADAMTS16 MUT2-Luc. HEK293T, A549, and H1975 cells were transfected with these constructs and pRL-TK plasmids. Luciferase activity was detected via a dual luciferase reporter assay kit (Vazyme, Nanjing, China) to determine firefly/renilla luciferase activity.

**ELISA**

Culture supernatants from A549 and H1975 cells were collected after transfection with OE-ADAMTS16 or vector plasmids for 48 h and subjected to ELISA to quantify the concentrations of total and active TGF-β1. This analysis was carried out via a human TGF-β1 ELISA Kit (R&D Systems, Minneapolis, MN, USA). Additionally, the same ELISA kit was used to quantify the plasma TGF-β1 concentration in LUAD patients.

**Bioinformatic analyses**

Transcriptome data from the TCGA-LUAD and GSE43458 cohorts were downloaded from The Cancer Genome Atlas (TCGA) (<https://gdc-portal.nci.nih.gov/>) and the Gene Expression Omnibus (GEO) database (https://www.ncbi.nlm.nih.gov/geo/), respectively. The proteome data of CPTAC-LUAD were downloaded from the Clinical Proteomic Tumor Analysis Consortium (CPTAC) database (<https://pdc.cancer.gov/pdc/browse>). Kaplan–Meier survival curves of patients with ADAMTS16 expression in the GEO database were obtained from the Kaplan–Meier plotter (<http://kmplot.com/analysis/>). Differentially expressed genes between tumor and normal lung tissues were identified via the "limma" package in R with the threshold of a false discovery rate (FDR) <0.05. SOX4-binding sites in the ADAMTS16 promoter were predicted via the JASPAR database (<https://jaspar.genereg.net>). Gene set enrichment analysis (GSEA) was performed using the TCGA-LUAD dataset with GSEA software (version 4.3.2). The codes used in this study is available from the corresponding author on reasonable request.
