# Supplementary material for: ADAMTS16 drives epithelial-mesenchymal transition and metastasis through a feedback loop upon TGF-β1 activation in lung adenocarcinoma
Source: Cell Death Dis. 2024 Nov 17;15(11):837. doi: 10.1038/s41419-024-07226-z (PMC11570625; doi:10.1038/s41419-024-07226-z)
Supplement: Supplementary file 3 — Supplementary figures(1–8) and tables(1,3–8) [file 41419_2024_7226_MOESM3_ESM.pdf]

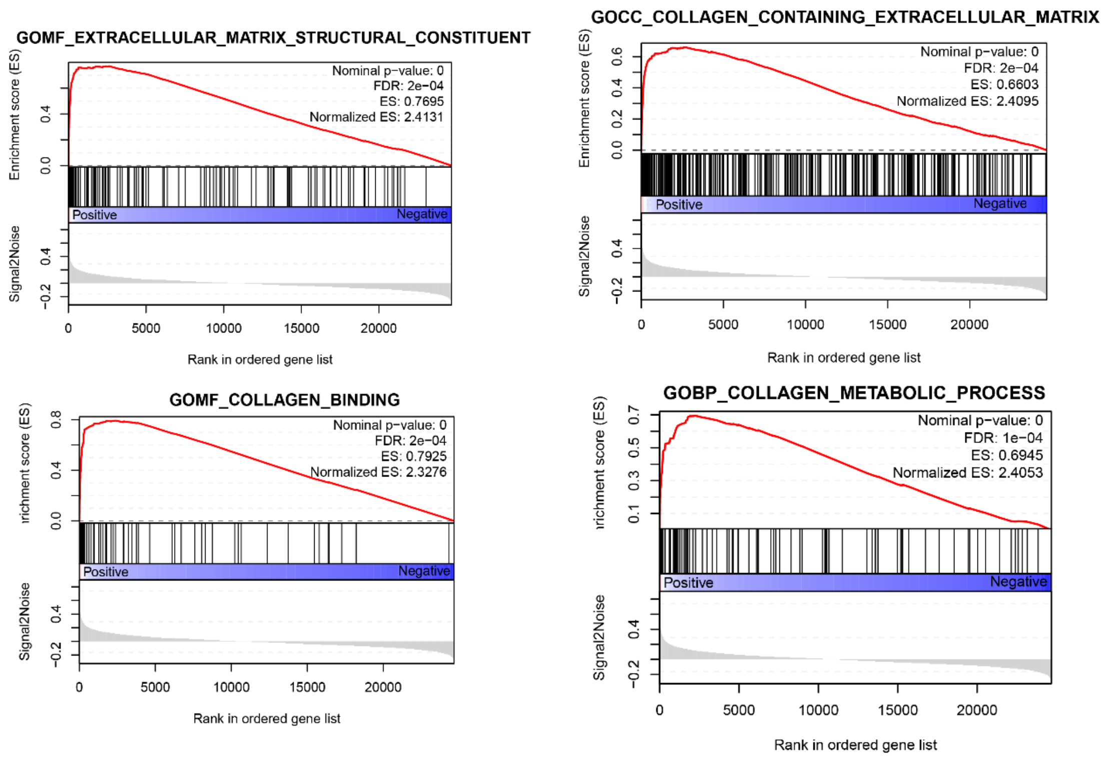

Supplementary Figure 1 GSEA plots indicating associations between expression of ADAMTS16 and ECM-related processes.

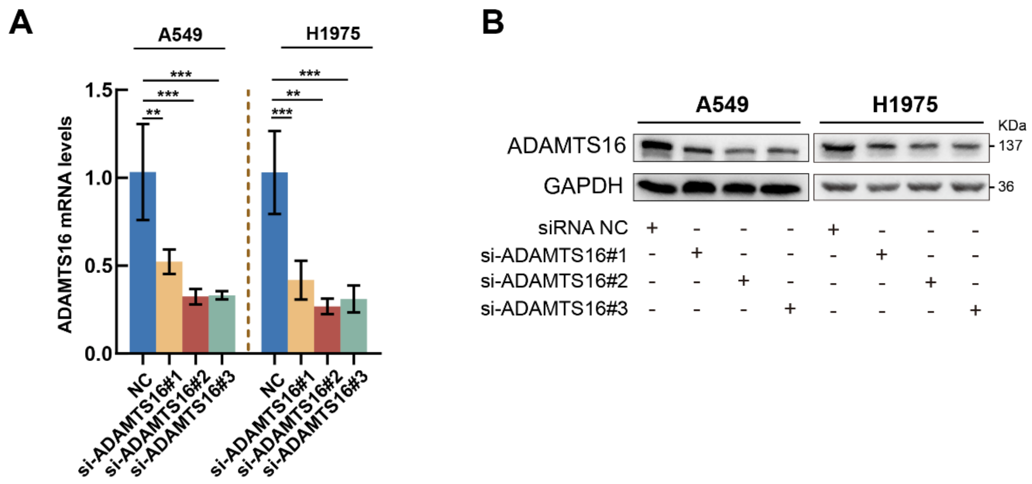

Supplementary Figure 2 Efficiency of ADAMTS16 knockdown by siRNA.

(A) Efficiency of ADAMTS16 knockdown, as indicated by qRT-PCR. **(B)** The efficiency of ADAMTS16 knockdown determined by immunoblotting. Data was expressed as mean ± SEM (n = 3 for each group) and was compared using Student's t-test. *P<0.05; **P<0.01; ***P<0.001;ns, not significant.

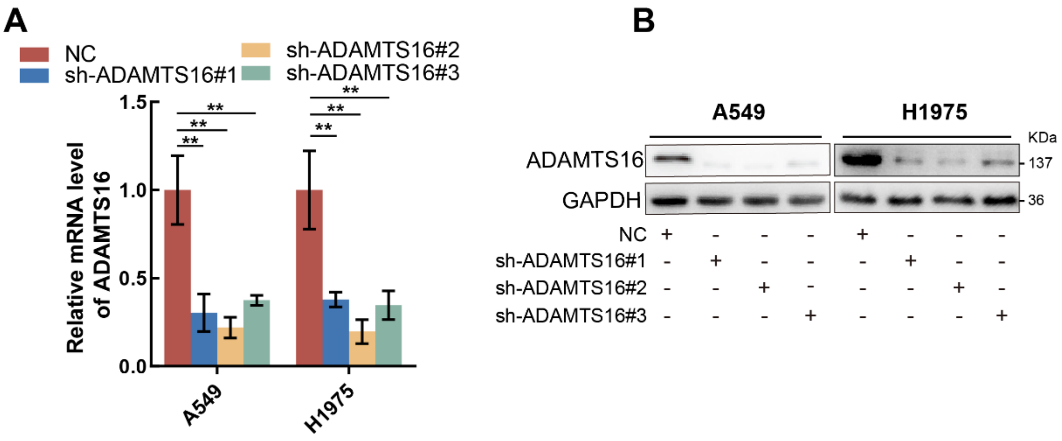

Supplementary Figure 3 Efficiency of ADAMTS16 knockdown by shRNA. (A) Efficiency of ADAMTS16 knockdown, as indicated by qRT-PCR. **(B)** The efficiency of ADAMTS16 knockdown determined by immunoblotting. Data was expressed as mean ± SEM (n = 3 for each group) and was compared using Student's t-test. *P<0.05; **P<0.01; ***P<0.001;ns, not significant.

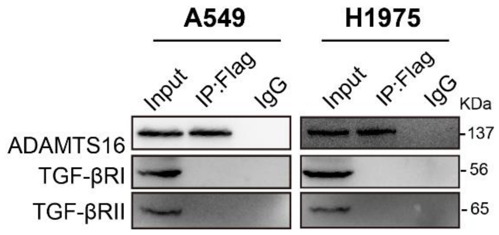

Supplementary Figure 4 Immunoprecipitation showing the interaction between ADAMTS16 and TGF-βRI/TGF-βRII.

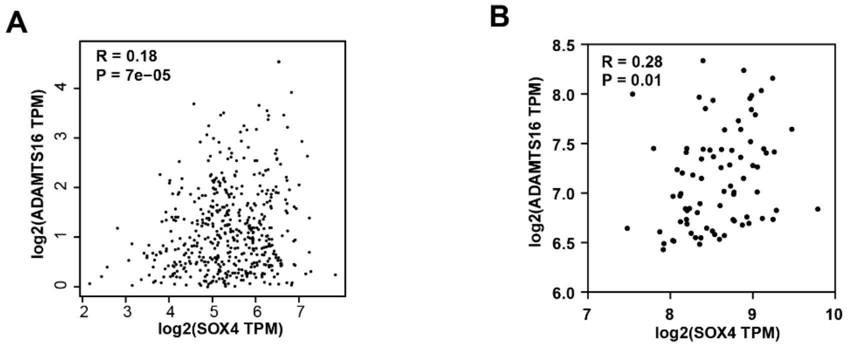

Supplementary Figure 5

Correlation between the expression of SOX4 and ADAMTS16

(A, B) Correlations between the expression of SOX4 and ADAMTS16 in LUAD samples in the TCGA (n=535) and GEO (n=110) databases. Data was analyzed by Pearson correlation analysis.

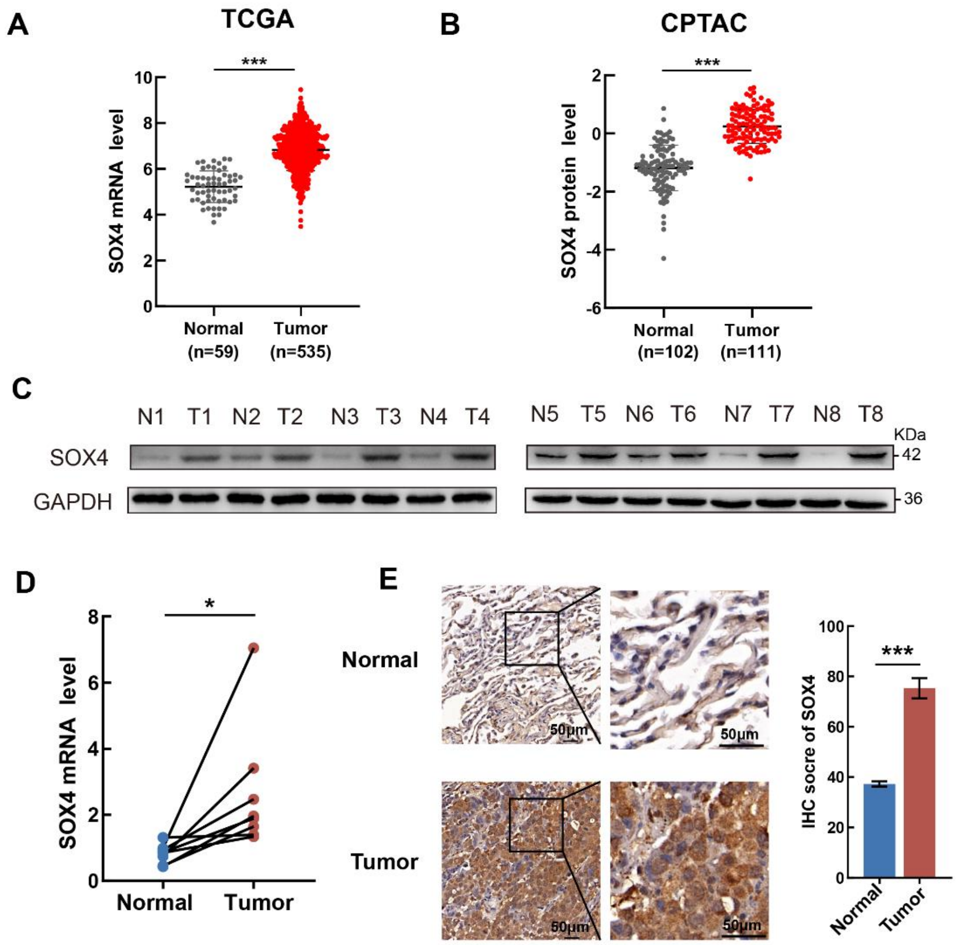

Supplementary Figure 6 Expression of SOX4 between LUAD tissues and normal lung tissues. **(A)** Expression of SOX4 in LUAD and normal lung tissues in the TCGA-LUAD cohort. **(B)** Expression of SOX4 in LUAD and normal lung tissues in the CPTAC-LUAD cohort. **(C)** Expression of SOX4 between LUAD and normal lung tissues in clinical samples indicated by immunoblotting. **(D)** Expression of SOX4 between LUAD and normal lung tissues in clinical samples indicated by qRT-PCR. **(E)** Expression of SOX4 in LUAD and normal lung tissues in clinical samples indicated by IHC. Data was expressed as mean ± SEM (n = 3 for each group) and was compared using Student's t-test. *P<0.05; **P<0.01; ***P<0.001;ns, not significant.

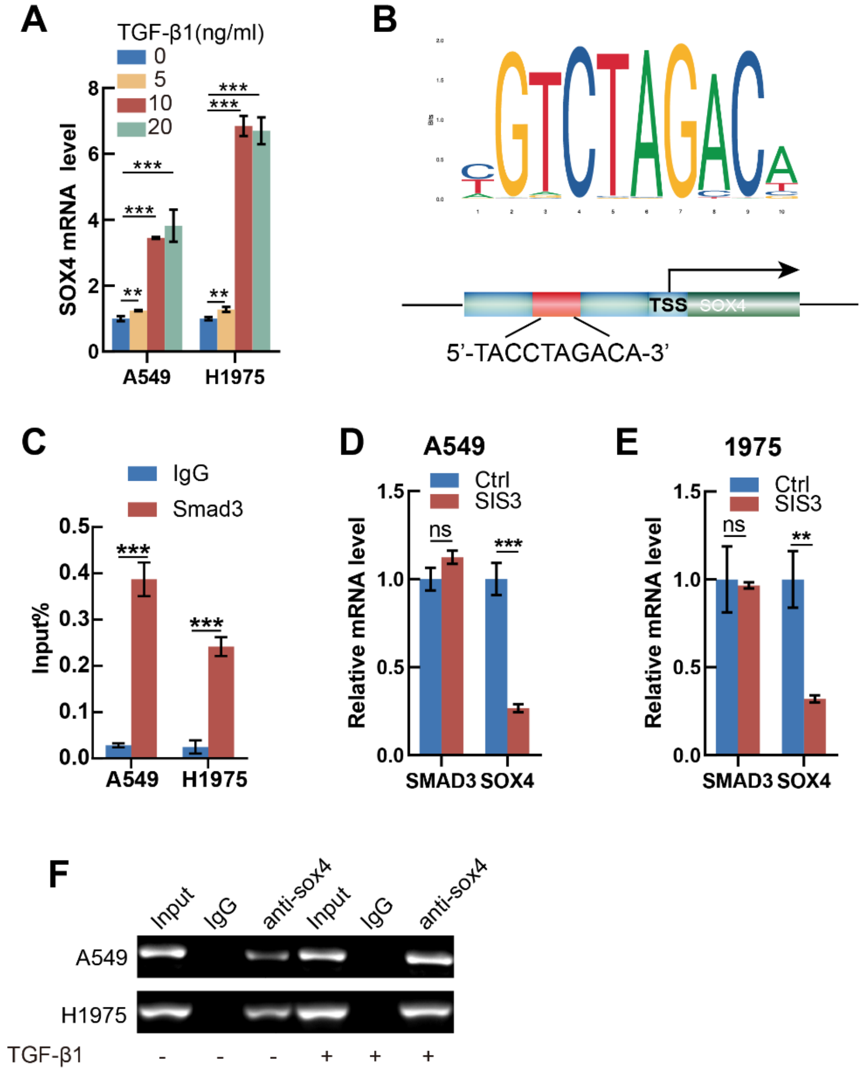

Supplementary Figure 7 TGF-β1 regulated SOX4 via Smad3. (A) Effect of TGF-β1 stimulation on mRNA expression of SOX4. **(B)** The Smad3-binding sequences in the promoter of SOX4 acquired from JASPAR. **(C)** ChIP-qPCR results indicating the interaction between Smad3 and the promoter of SOX4. **(D-E)** Effect of SIS3 on mRNA expression of SOX4. **(F)** ChIP assay indicating the effect of TGF-β1 on the interaction between SOX4 and the promoter of ADAMTS16. Data was expressed as mean ± SEM (n = 3 for each group) and was compared using Student's t-test. *P<0.05; **P<0.01; ***P<0.001;ns, not significant.

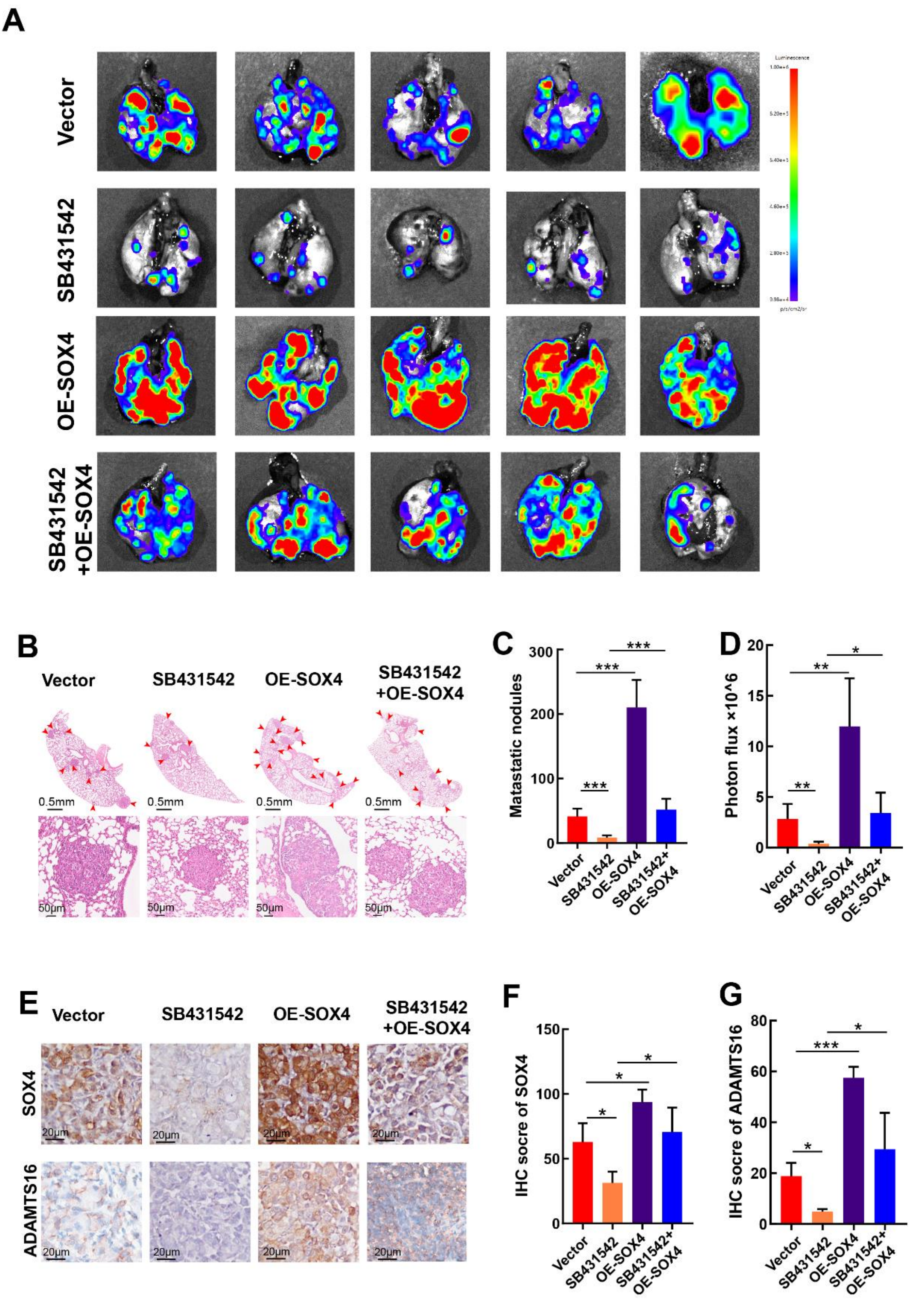

Supplementary Figure 8 Effects of SOX4 and SB431542 on lung metastasis.

(**A**) Metastatic lesions in the lungs captured by bioluminescence imaging showing the

effects of SB431542 and OE-SOX4 on lung metastasis. (**B**) H&E staining of lung metastatic lesions. **(C)** Counts of metastatic nodules on the surface of the lungs in each group. **(D)** Luciferase activity of the lungs in each group. (**E-G**) IHC staining of SOX4 and ADAMTS16 in lung metastatic lesions. Data was expressed as mean ± SEM (n=5 for each group) and was compared using Student's t-test. *P<0.05; **P<0.01; ***P<0.001;ns, not significant.

Supplementary Table1: Summary of proteins datasets used in this study

Study PMID	Sample size	Number of proteins	Platform
37996402	70,877	738	Olink
37794186	54,306	1,463	Olink
34857953	35,559	4,719	SomaLogic
33067605	30,000	90	Olink
34648354	10,708	4,775	SomaLogic
30111768	6,861	71	xMAP
29875488	3,301	2,995	SomaLogic
28240269	1,000	1124	SomaLogic

Supplementary Table 3 Sequences of siRNAs

Gene	Sequences (5'-3')
Human ADAMTS16-si1	GGACUUCUGUUUCUAUCAATT<br>UUGAUAGAAACAGAAGUCCTT
Human ADAMTS16-si2	GCAGACCACACCUUAAGUATT<br>UACUUAAGGUGUGGUCUGCTT
Human ADAMTS16-si3	CCGCCAGUAUCUACACAAATT<br>UUUGUGUAGAUACUGGCGGTT
Human SOX4-si1	GCAAACCAACAAUGCCGAGTT<br>CUCGGCAUUGUUGGUUUGCTT
Human SOX4-si2	GCGACAAGAUCCCUUUCAUTT<br>AUGAAAGGGAUCUUGUCGCTT
Human SOX4-si3	ACCACCACUCGCUGUACAATT<br>UUGUACAGCGAGUGGUGGUTT

Supplementary Table 4 Sequences of shRNAs

ID	Sequence (5'-3')
Human sh-ADAMTS16#1	CCTGTGAAGGAATACAAGTAT
Human sh-ADAMTS16#2	GACTACAGACGGTCCTATAAT
Human sh-ADAMTS16#3	GCCGCCAGTATCTACACAAAT
Human sh-NC	GTTCTCCGAACGTGTCACGT

Supplementary Table 5 Clinical characteristics of clinical samples

ID	Sex	Age	Diagnosis	stage
1	Female	64	LUAD	I
2	Female	46	LUAD	I
3	Male	59	LUAD	I
4	Male	58	LUAD	III
5	Male	66	LUAD	I
6	Female	58	LUAD	III
7	Male	54	LUAD	I
8	Female	64	LUAD	II
9	Female	46	LUAD	III
10	Male	69	LUAD	IV
11	Male	65	LUAD	I
12	Female	77	LUAD	I

Supplementary Table 6 Clinical characteristics of samples in tissue microarray

Variables	Total	ADAMTS16 Negative(%)	ADAMTS16 Positive(%)
Age			
<65	55(62.5%)	21(53.85%)	34(69.39%)
≥65	33(37.5%)	18(46.15%)	15(30.61%)
Sex			
Female	41(46.59%)	16(41.03%)	25(51.02%)
Male	47(53.41%)	23(58.97%)	24(48.98%)
T			
1-2	78(88.64%)	35(89.74%)	43(87.76%)
3-4	10(11.36%)	4(10.26%)	6(12.24%)
N			
0-1	73(82.95%)	33(84.62%)	40(81.63%)
2-3	15(17.05%)	6(15.38%)	9(18.37%)
M			
0	88(100%)	39(100%)	49(100%)
1	0(0%)	0(0%)	0(0%)
Stage			
I-II	68(77.27%)	29(74.36%)	39(79.59%)
III-IV	20(22.73%)	10(25.64%)	10(20.41%)

Supplementary Table 7 Primers used for qRT-PCR

Gene		Sequences
ADAMTS16	Forward	TGCCCGTGTCCGAGGTTGAG
	Reverse	CACAATAAAGCCAGGAGCCACTAGG
SOX4	Forward	ACCAACAATGCCGAGAACACG
	Reverse	CTCGATCTGCGACCACACCAT
E-cadherin	Forward	GCCATCGCTTACACCATCCTCAG
	Reverse	CTCTCTCGGTCCAGCCCAGTG
Vimentin	Forward	ACCAGCCGCAGCCTCTACG
	Reverse	AGCGAGAAGTCCACCGAGTCC
ZO-1	Forward	AACTGGGCTCTTGGCTTGCTATTC
	Reverse	TCCAGAAGTCAGCACGGTCTCC
TGFB1	Forward	ACCTGCCACAGATCCCCTAT
	Reverse	CCGGTAGTGAACCCGTTGAT
Smad3	Forward	TAATTTATTGCCGCCGCTCG
	Reverse	GGGGTGGGGTCTCTGGAATA
GAPDH	Forward	GGAGTCCACTGGCGTCTTCA
	Reverse	GTCATGAGTCCTTCCACGATACC

Supplementary Table 8 Primers used for chip-qPCR

Site		Sequences
ADAMTS16-siteA	Forward	ACTAATCTGATGGGGACG
	Reverse	GATTGGTAGAGCCGAGTG
ADAMTS16-siteB	Forward	GCTCAGGTCCCCTTCCAT
	Reverse	GCGAGACTCCGTCTCAAA
ADAMTS16-siteC	Forward	TCTGGCGCTGCCCGCTGTCT
	Reverse	AGAGGAGGGGAGGAGGTG
SOX4-site	Forward	ATCTGTGCTGTACCTAGAGTTGTT
	Reverse	TGTAAAGGGGAAGAGAAAGCCAC
