# Supplementary material for: ADAMTS16 drives epithelial-mesenchymal transition and metastasis through a feedback loop upon TGF-β1 activation in lung adenocarcinoma
Source: Cell Death Dis. 2024 Nov 17;15(11):837. doi: 10.1038/s41419-024-07226-z (PMC11570625; doi:10.1038/s41419-024-07226-z)

Figure 2 D

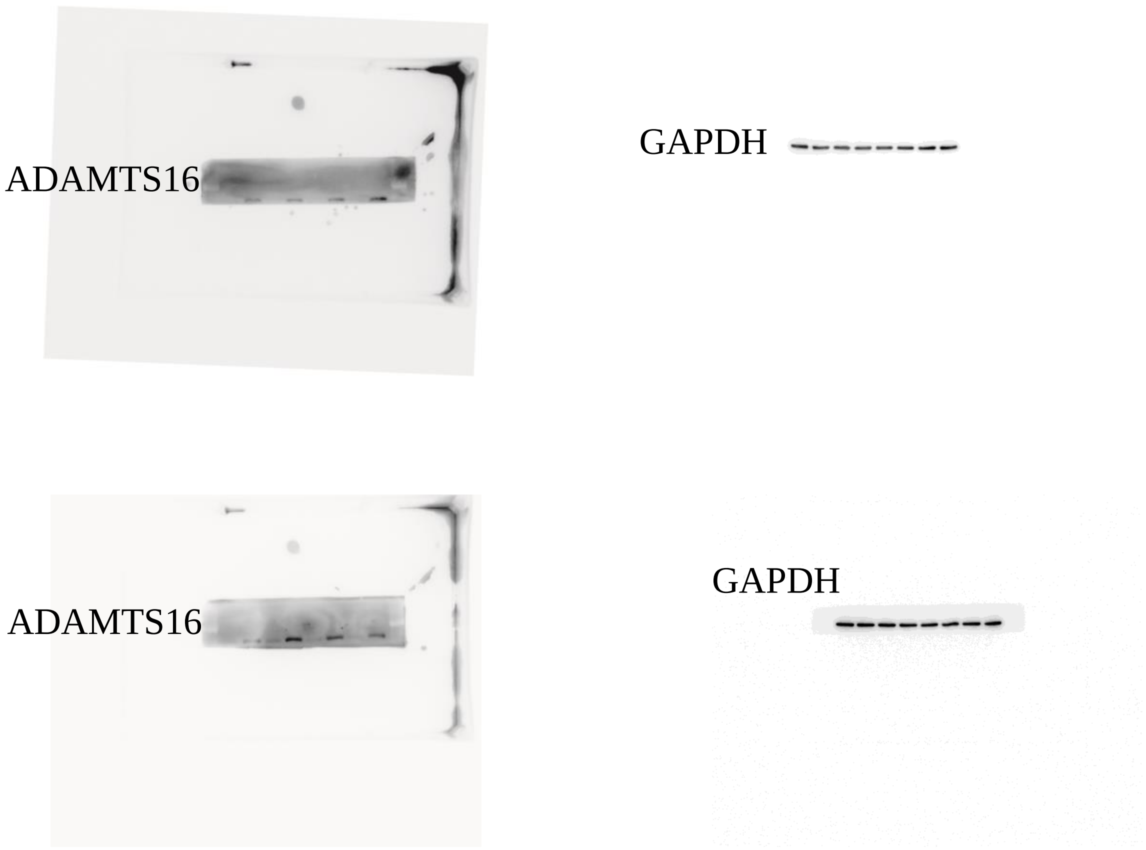

- Figure 3 B-A549 (n=3)

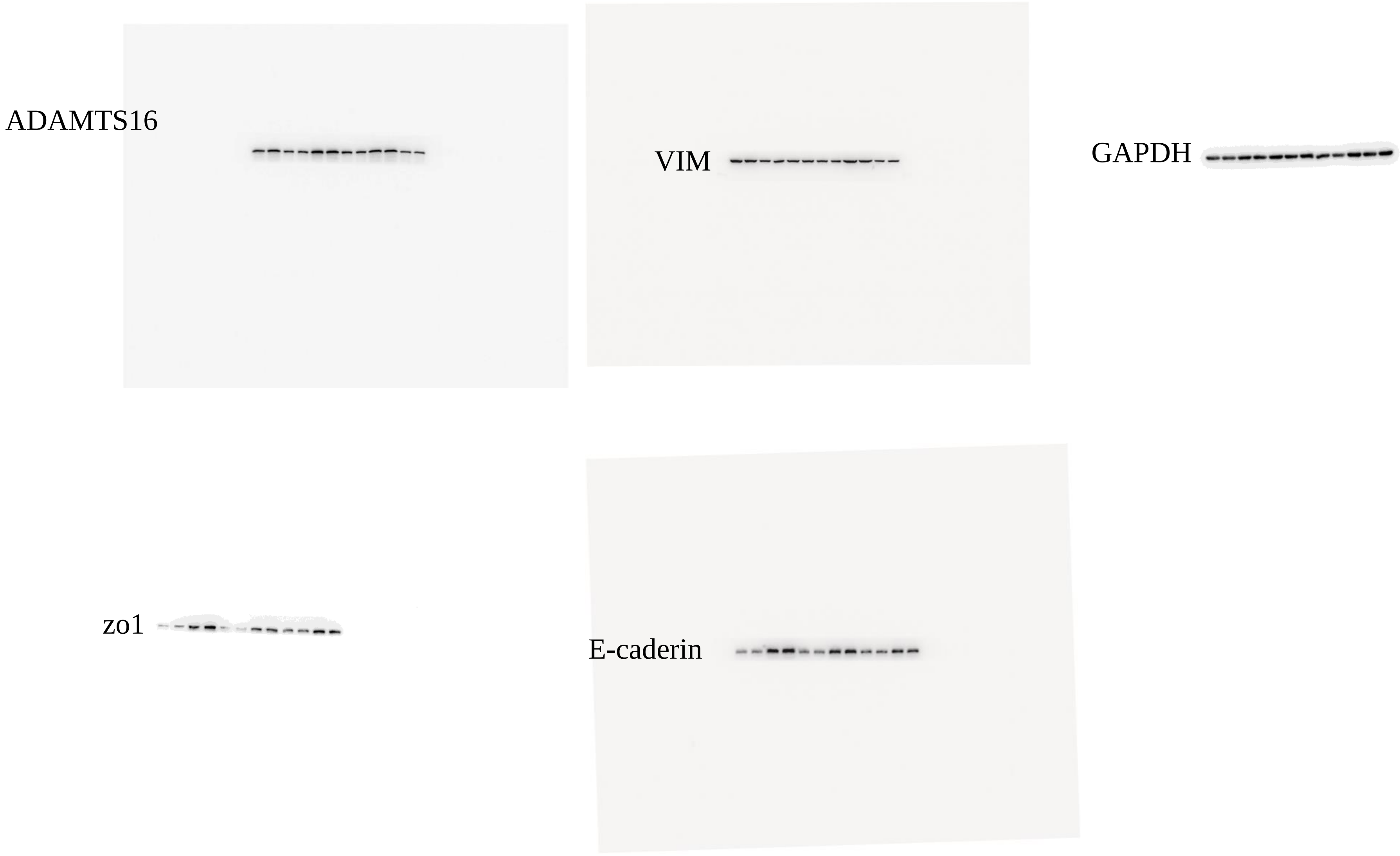

- Figure 3 B-H1975 (n=3)

ADAMTS16

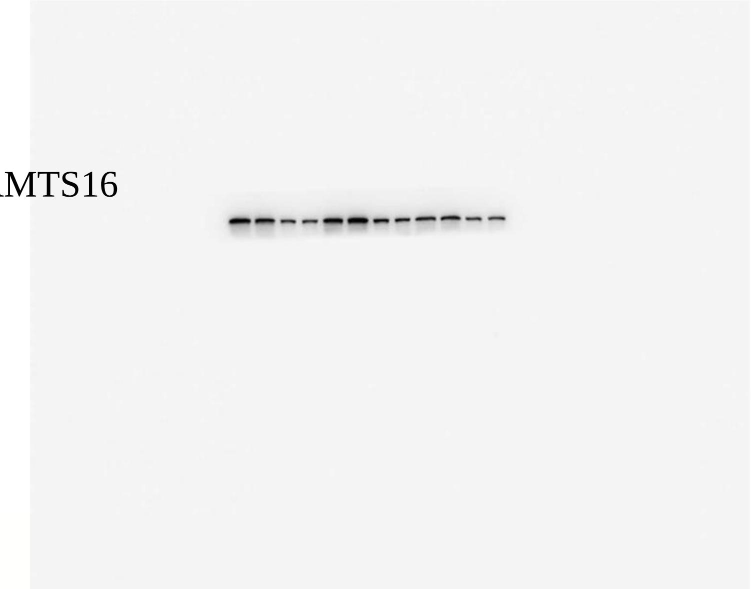

GAPDH

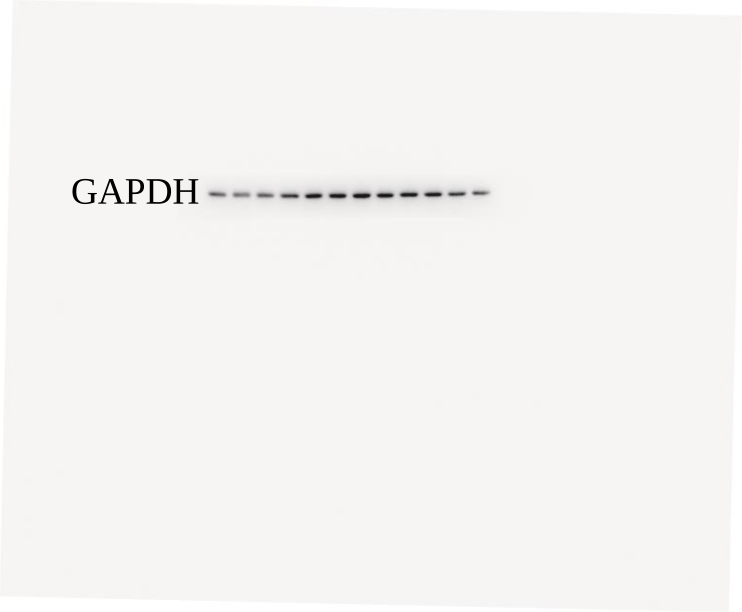

E-caderin

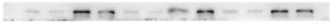

ZO1

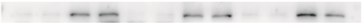

VIM

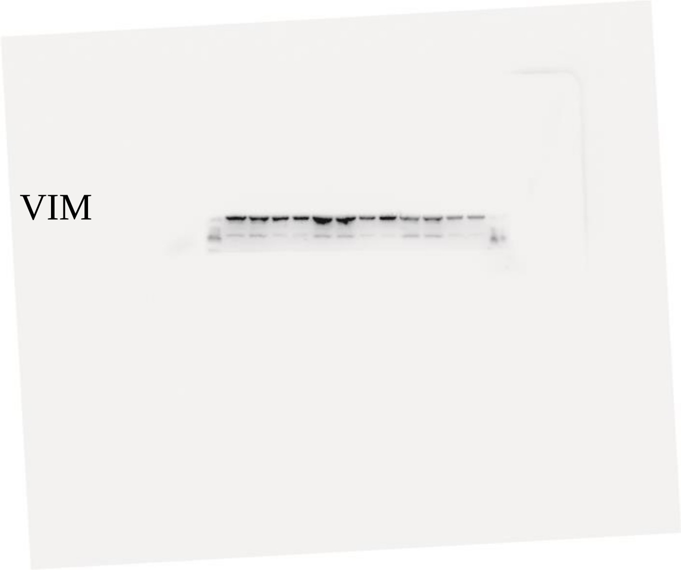

• Figure 5 C-A549

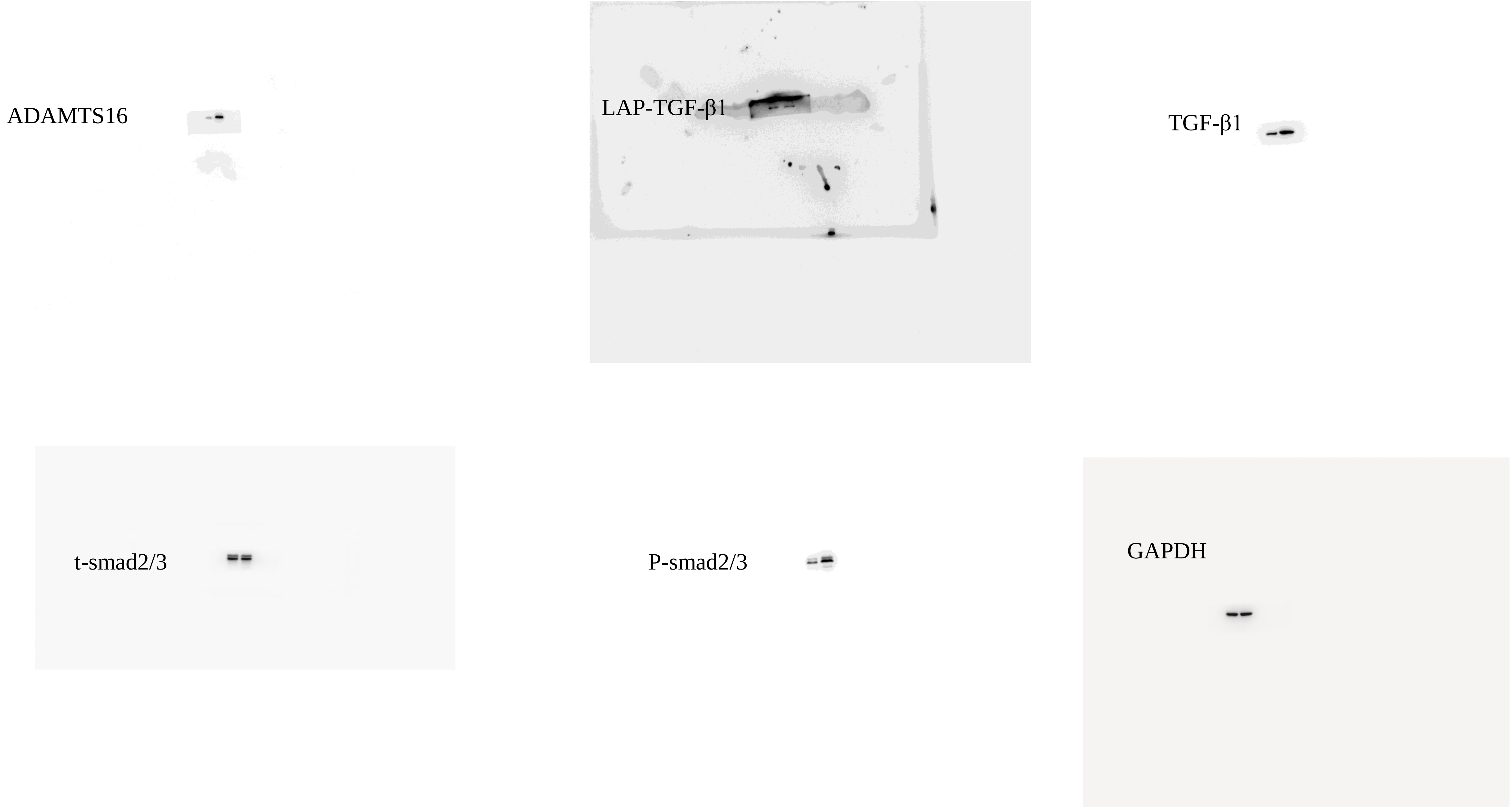

- Figure 5 C-H1975

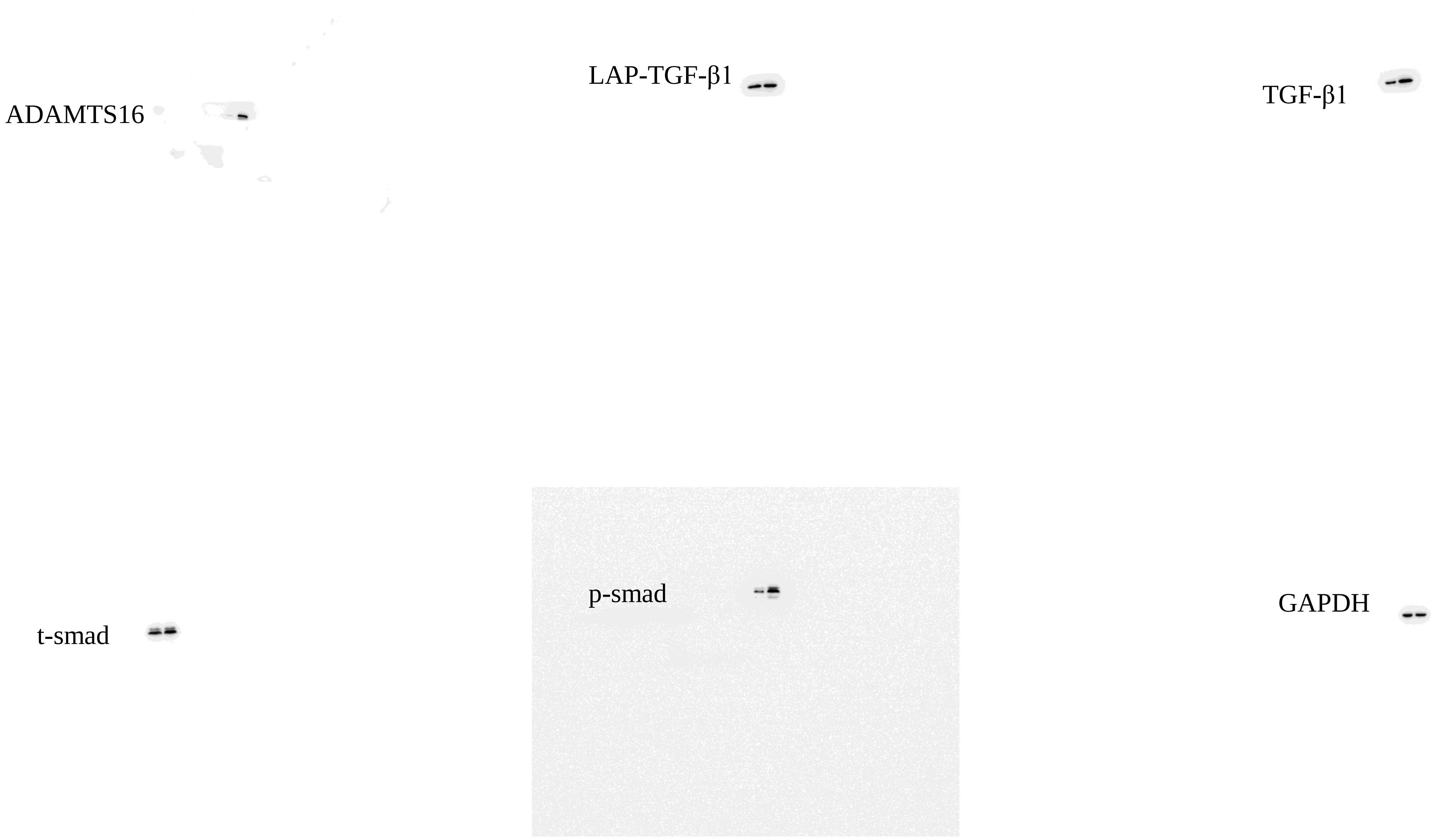

- Figure 5 D-A549 n=6

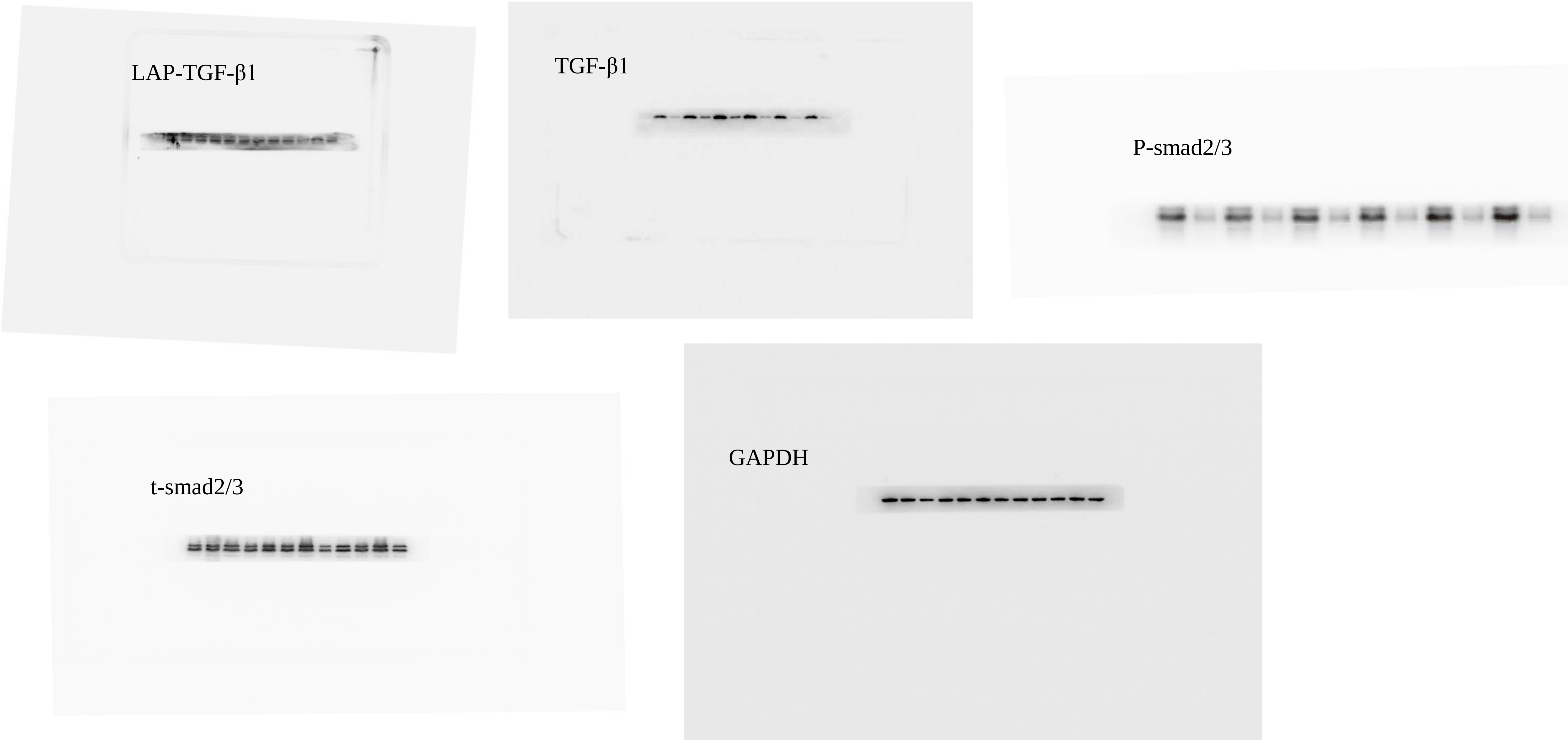

- Figure 5 D-H1975 n=6

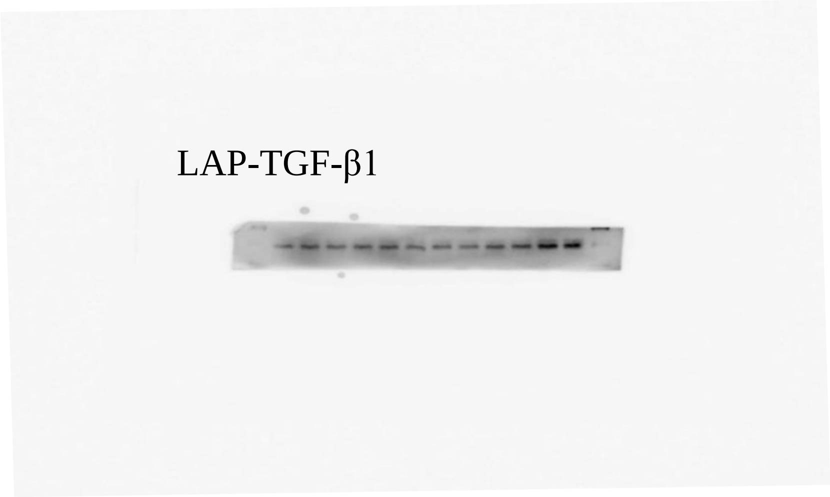

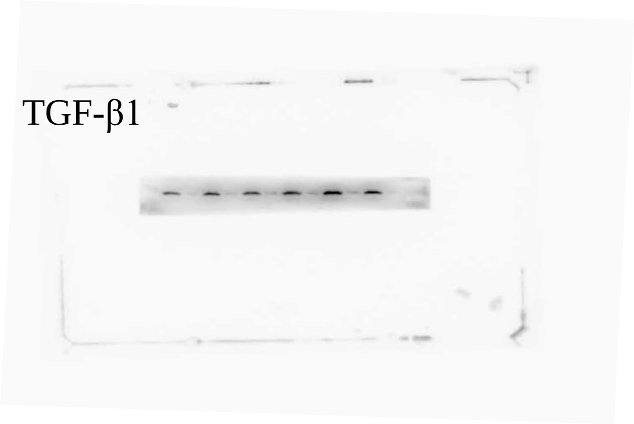

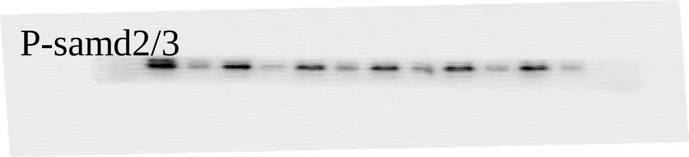

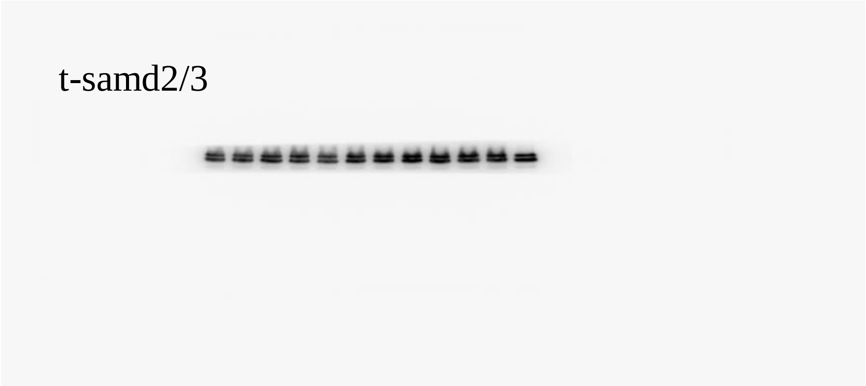

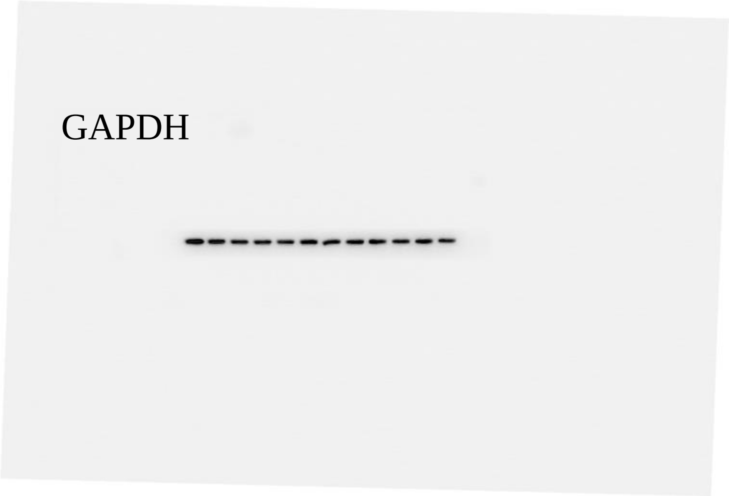

Figure 5F

A549

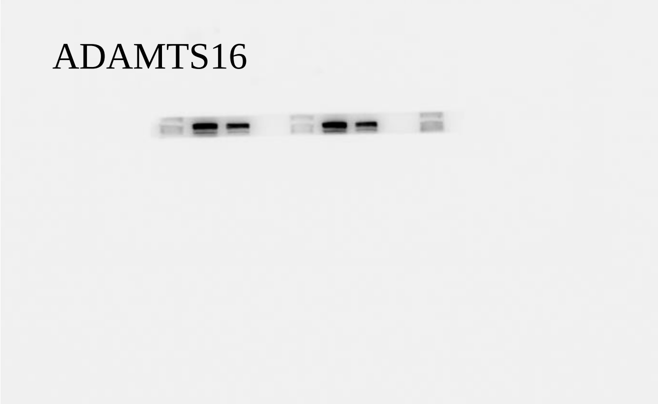

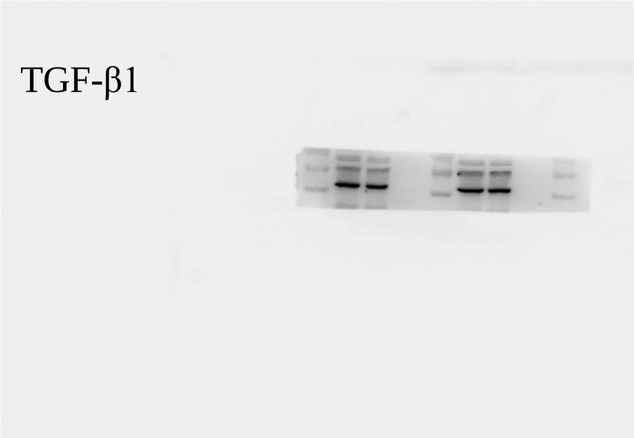

H1975

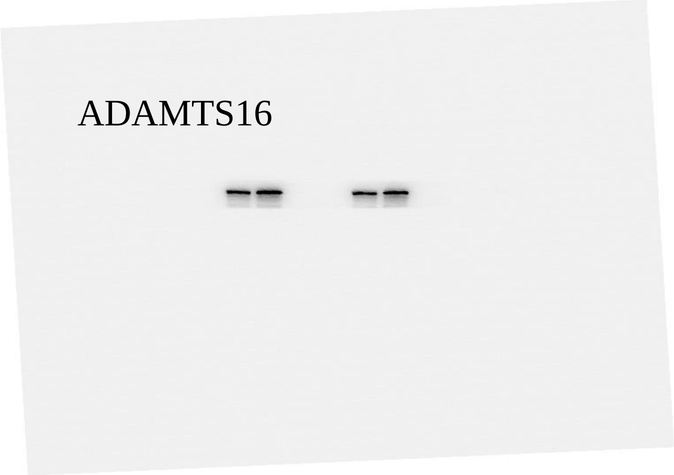

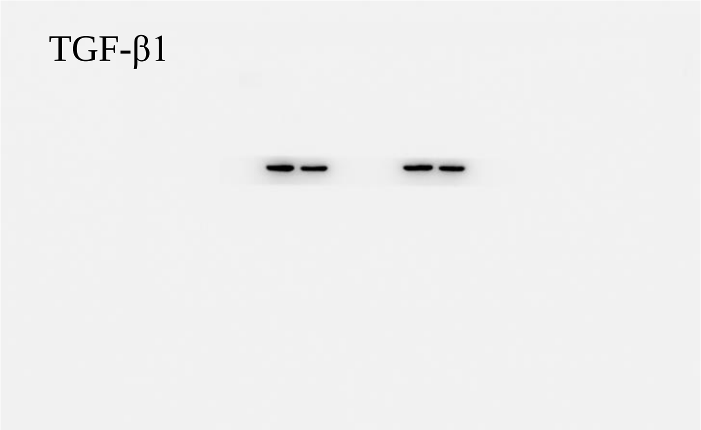

- Figure 6 F (n=3)

A549

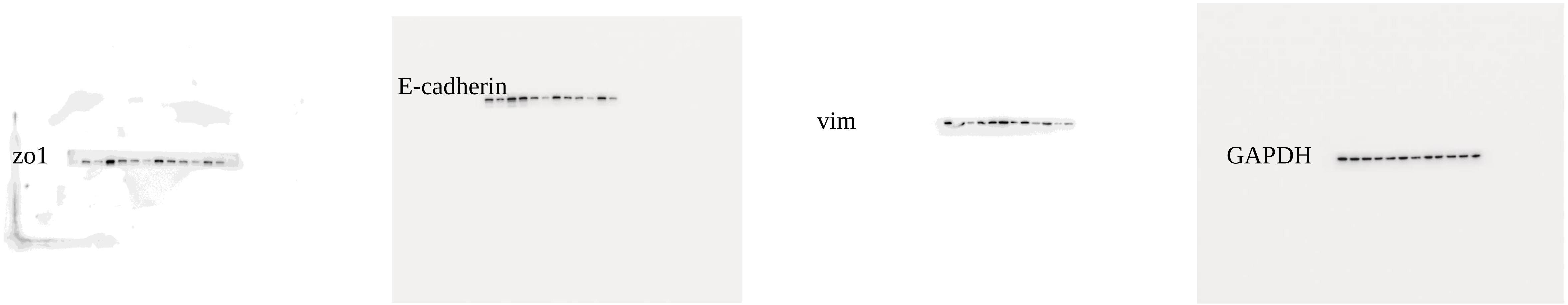

H1975

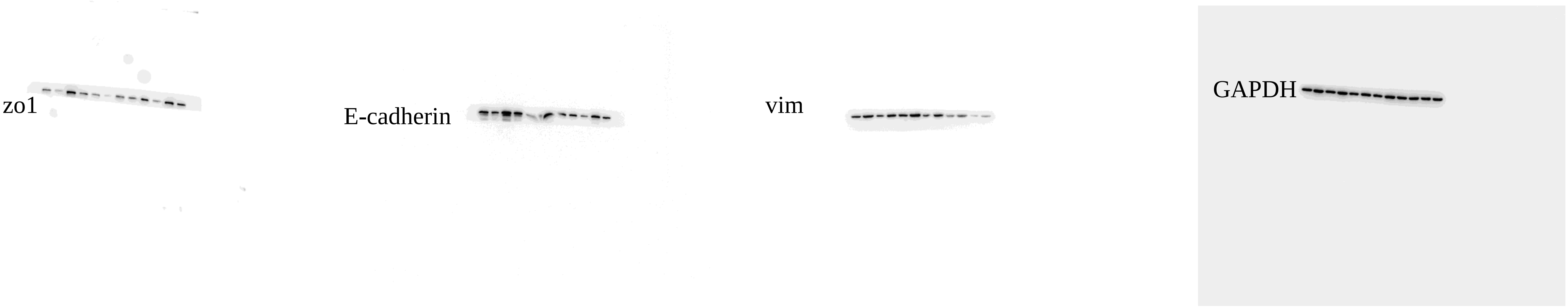

- Figure 8A

A549

SOX4

GAPDH

H1975

SOX4

GAPDH

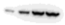

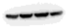

- Figure 8C

A549

p-smad3

t-smad3

SOX4

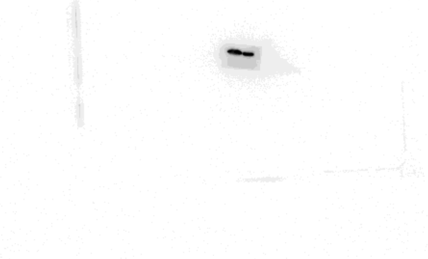

GAPDH

H1975

p-smad3

t-smad3

SOX4

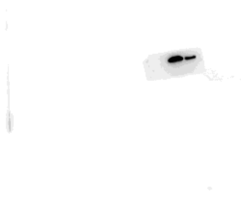

GAPDH

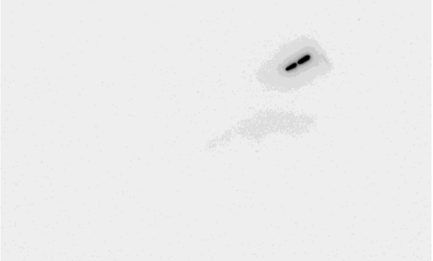

- Figure 8F(n=3)

A549

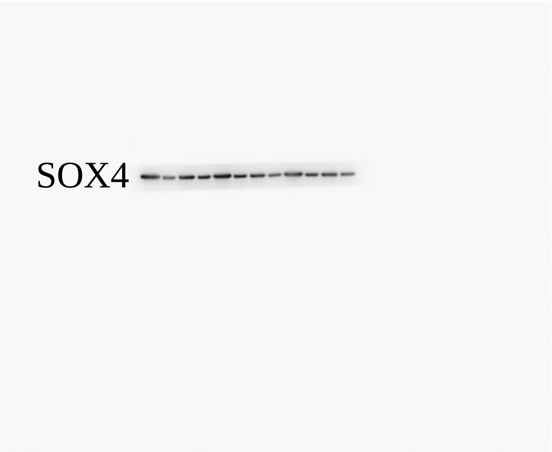

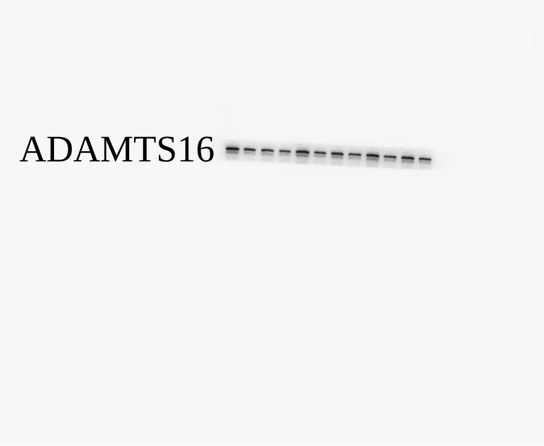

GAPDH

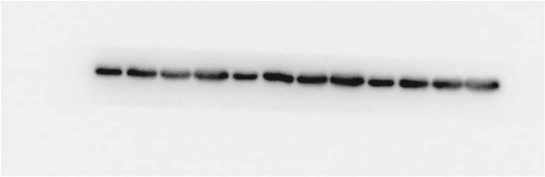

H1975

SOX4

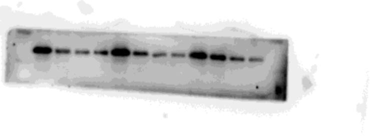

ADAMTS16

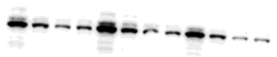

GAPDH

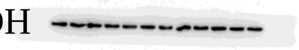

- Figure 8K-A549 (n=3)

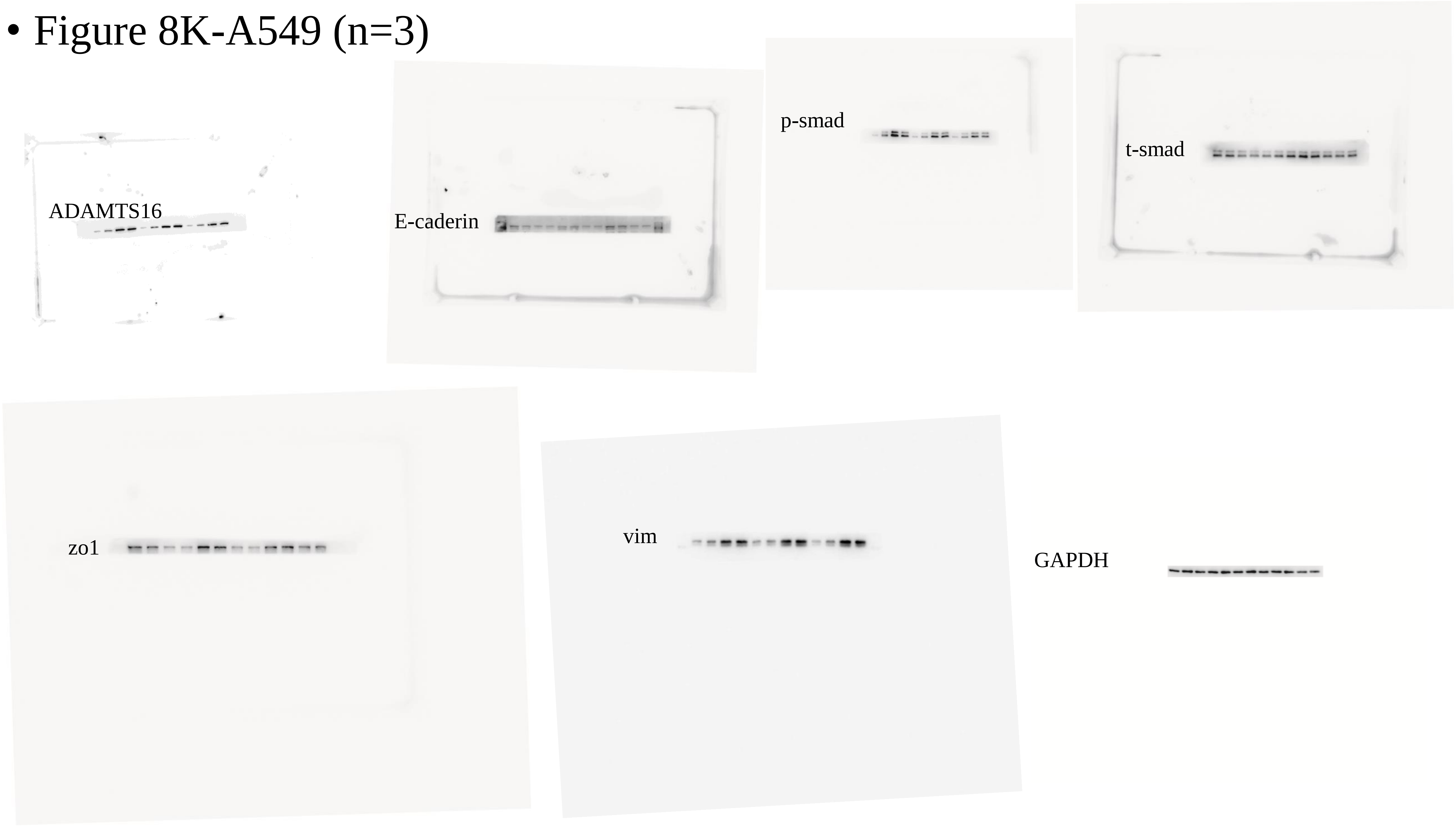

- Figure 8K-H1975 (n=3)

ADAMTS16

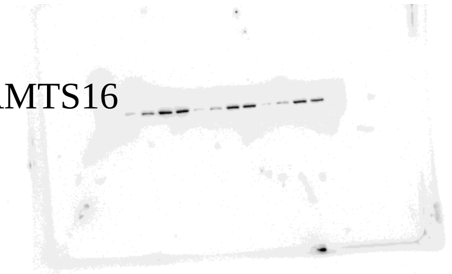

E-caderin

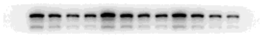

zo1

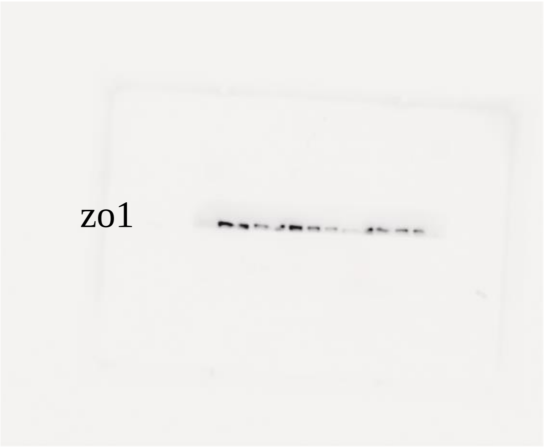

p-smad

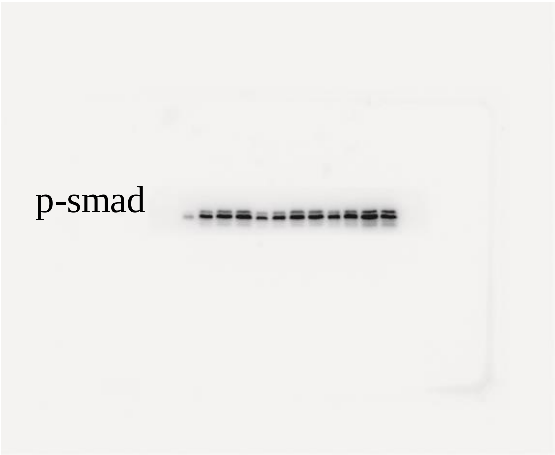

t-smad

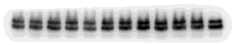

vim

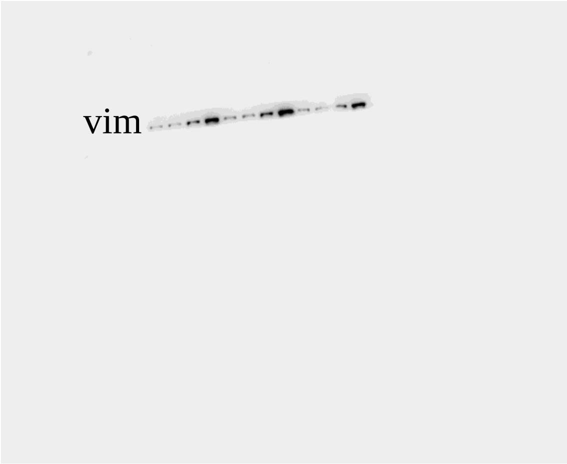

GAPDH

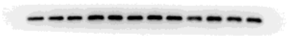

- # Figure 8L

A549

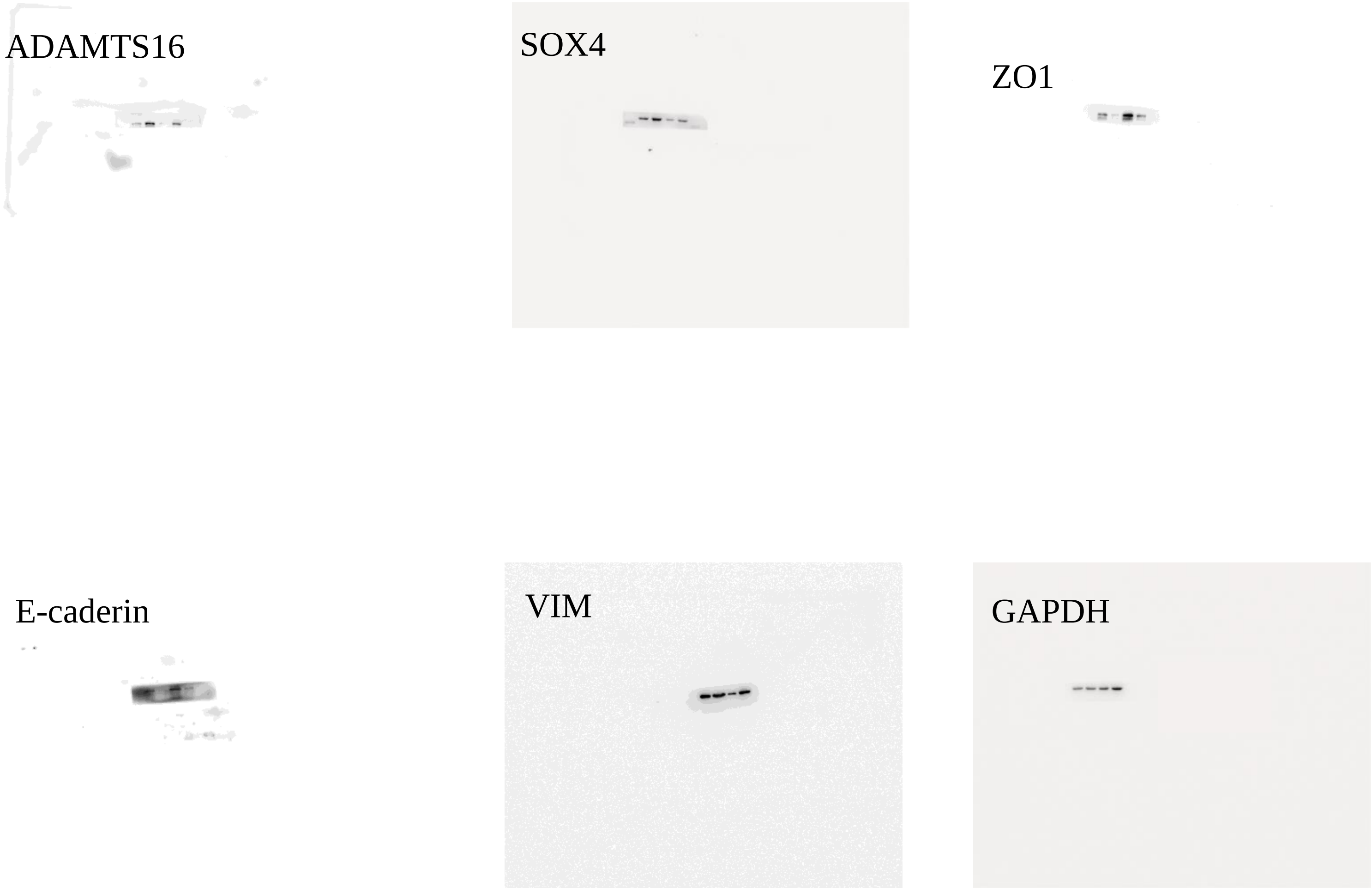

- Figure 8L

H1975

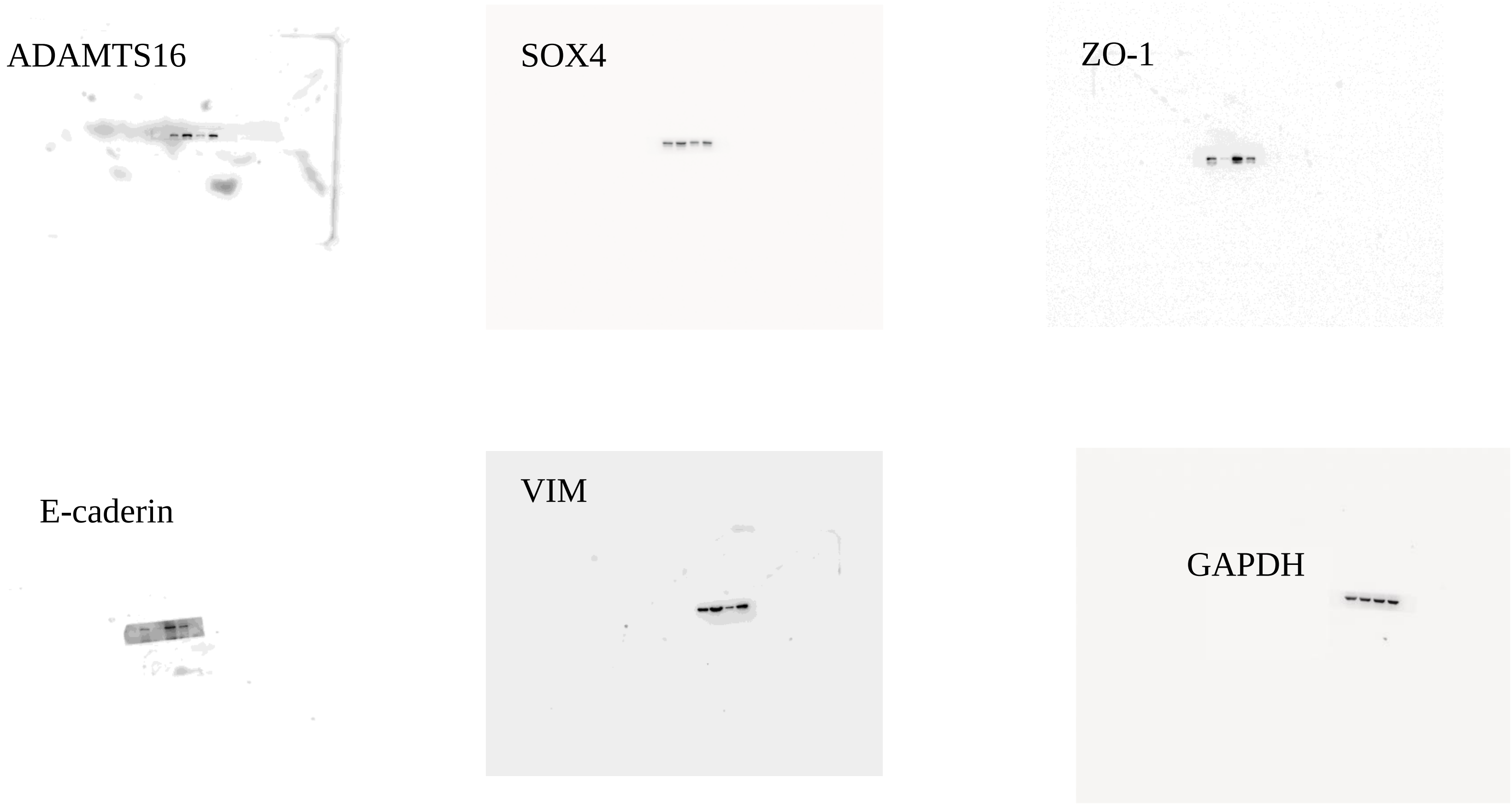

- Supplementary Figure 2

A549

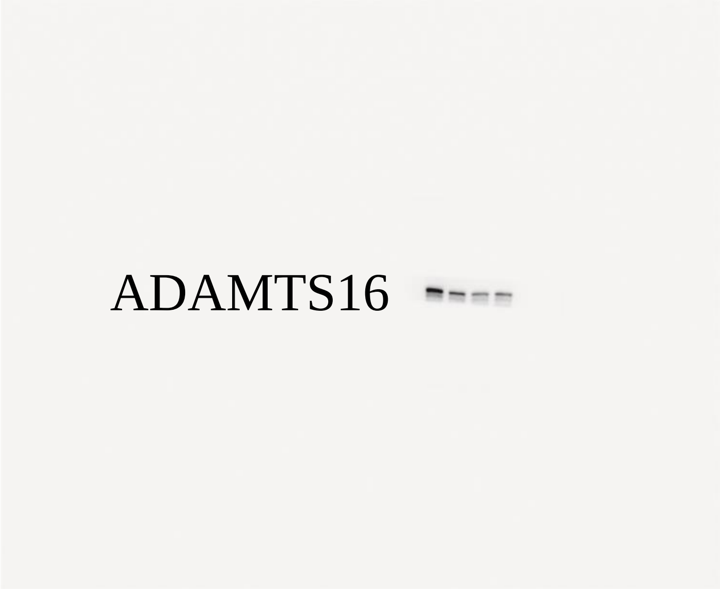

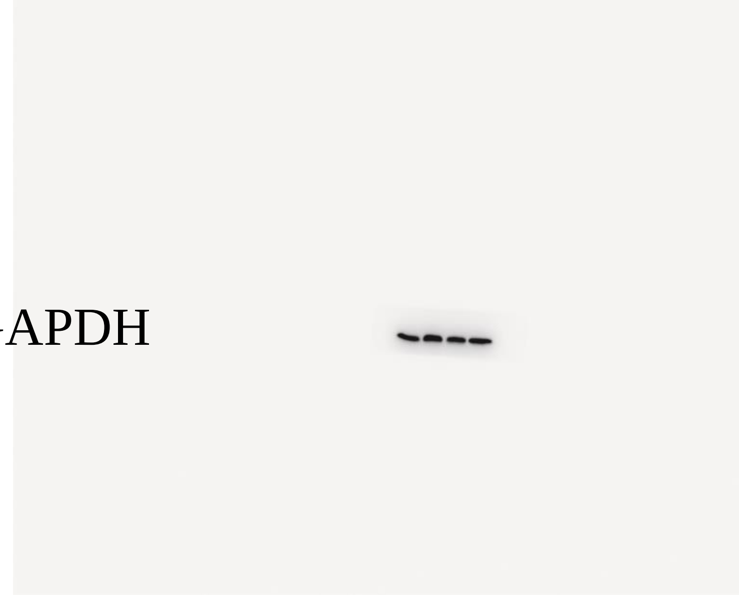

H1975

ADAMTS16

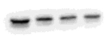

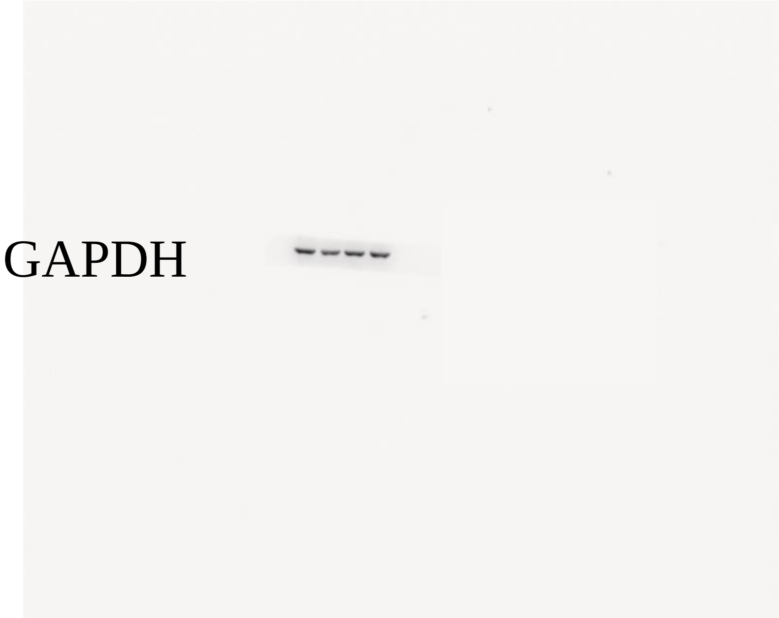

- Supplementary Figure 3 (n=3)

A549

ADAMTS16 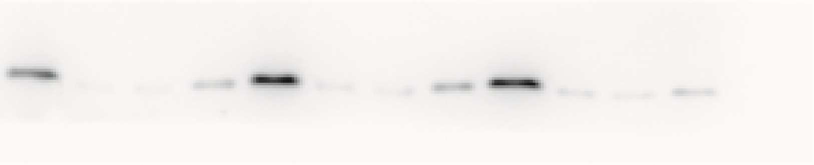

GAPDH 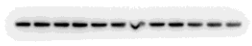

H1975

ADAMTS16 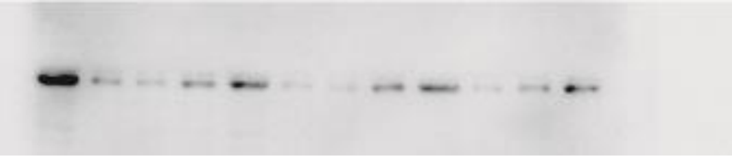

GAPDH 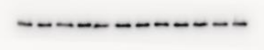

- Supplementary Figure 4

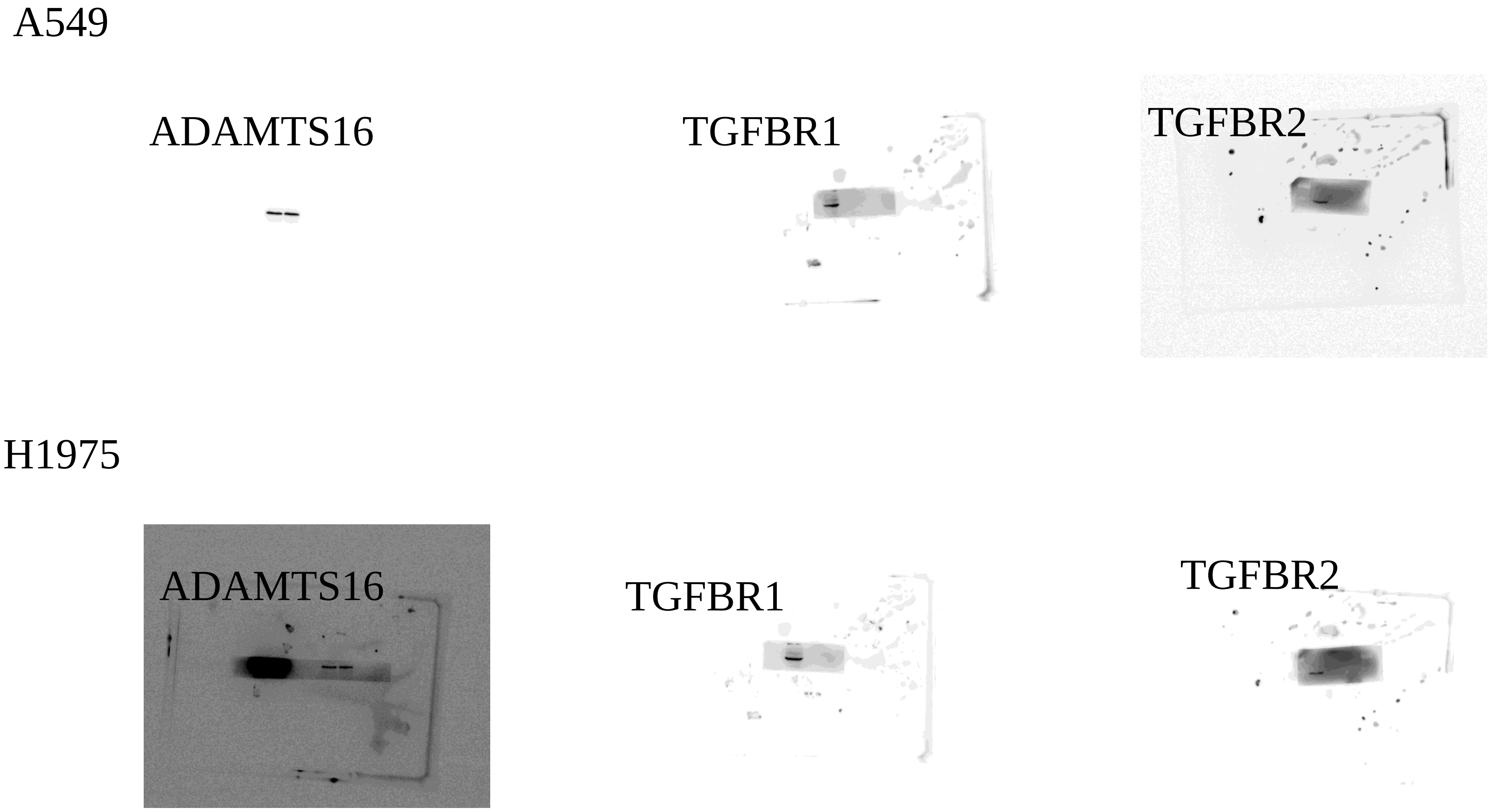

- Supplementary Figure 6

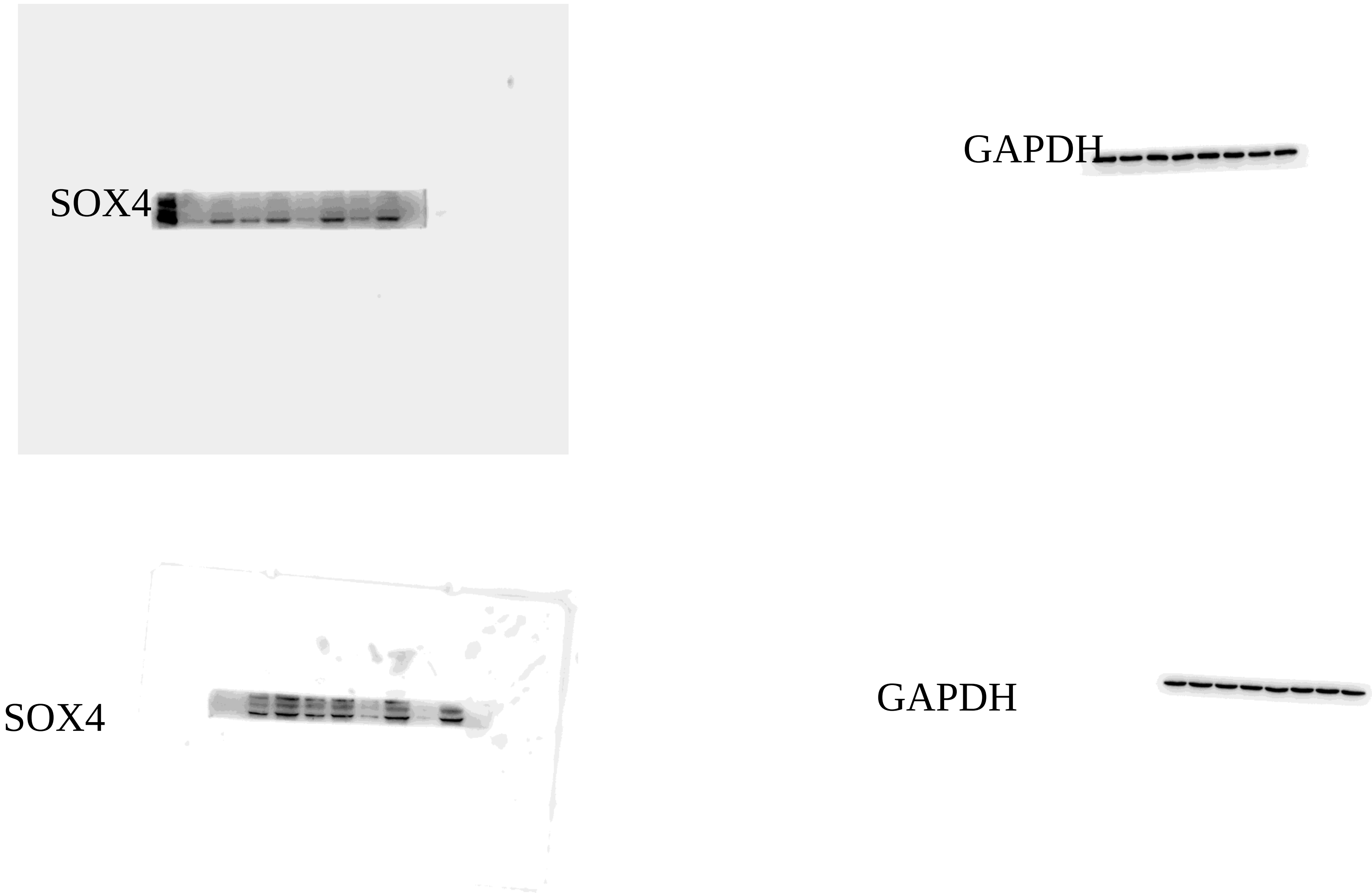

Supplement: Supplementary file 4 — full western blot [file 41419_2024_7226_MOESM4_ESM.pdf]
